# Polypyrimidine tract binding proteins PTBP1 and PTBP2 associate with distinct proteins and have distinct post-translational modifications in neuronal nuclear extract

**DOI:** 10.1371/journal.pone.0325143

**Published:** 2025-06-04

**Authors:** Michael E. Sullivan, Jacob A. Edberg, Christopher I. Nunez, Herbert L. Axelrod, Niroshika M. Keppetipola

**Affiliations:** Department of Chemistry and Biochemistry, California State University Fullerton, Fullerton, California, United States of America; Ural Federal University named after the first President of Russia B N Yeltsin Institute of Physics and Technology: Ural'skij federal'nyj universitet imeni pervogo Prezidenta Rossii B N El'cina Fiziko-tehnologiceskij institut, RUSSIAN FEDERATION

## Abstract

RNA binding proteins play an important role in regulating alternative pre-mRNA splicing and in turn cellular gene expression. Polypyrimidine tract binding proteins, PTBP1 and PTBP2, are paralogous RNA binding proteins that play a critical role in the process of neuronal differentiation and maturation; changes in the concentration of PTB proteins during neuronal development direct splicing changes in many transcripts that code for proteins critical for neuronal differentiation. PTBP1 can compensate for the loss of PTBP2 in some developmental contexts but not others signifying the paralogs have distinct functions. How two highly structurally similar proteins regulate different sets of neuronal exons is unclear and if known, will reveal how gene families evolved to achieve tissue-specific splicing and in turn, gene expression patterns. Here, we incubated PTBP1 and PTBP2 under splicing reaction conditions containing neuronal WERI retinoblastoma nuclear extract and probed for interacting partner proteins and chemical modifications via mass spectrometry. Our results reveal key differences in the kinds of proteins and processes the paralogs associate with under these conditions. Our data also highlight the presence of novel and distinct chemical modifications on the paralogs when incubated with neuronal nuclear extracts. Collectively, our study suggests a role for chemical modifications in regulating PTBP function in neuronal vs non-neuronal cells.

## Introduction

RNA binding proteins occur as gene families with paralogs sharing high primary and tertiary structural similarity. Yet these paralogs have non-overlapping tissue-specific expression patterns and can regulate distinct sets of target exons to elicit tissue-specific splicing programs. How structurally similar related members in a gene family exert tissue-specific splicing outcomes is not understood. Polypyrimidine Tract Binding Protein 1 (PTBP1) and its neuronal homolog Polypyrimidine Tract Binding Protein 2 (PTBP2) are paralogous RNA binding proteins that function as splicing regulatory proteins to either promote or inhibit the inclusion of cassette exons in the spliced mRNA [1]. PTBP1 is expressed near ubiquitously but is absent in neurons and myocytes while PTBP2 is expressed primarily in differentiating neurons and testis [2–6].

**Fig 1:**
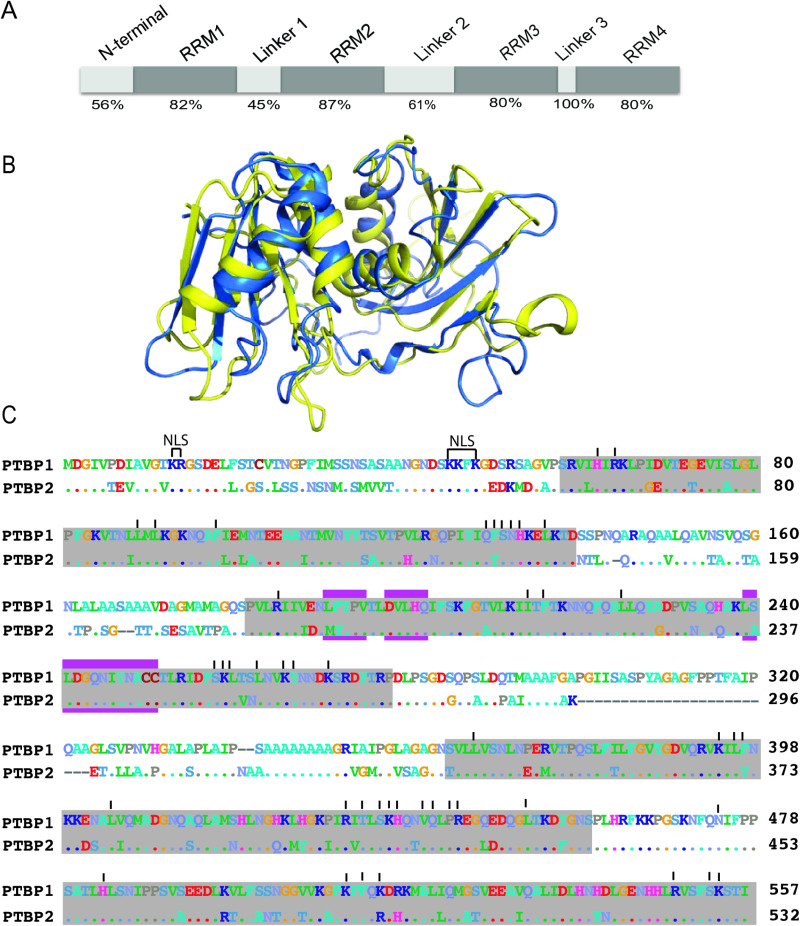
Structural organization of PTBP1 and PTBP2. **A)** Domain organization of PTB proteins. RRM-type RNA binding domains are indicated as dark grey rectangles. Linker regions connecting the RRMs and the N-terminal region are indicated in light gray rectangles. The amino acid sequence identity between the paralogs across each region is indicated at the bottom for each corresponding domain. B) ribbon representation comparisons of the representative solution structures of PTBP1 (blue, model 1, PDB ID 2MJU) and PTBP2 (yellow, PDB ID 2EVZ). The coordinates of the two were superimposed with the program GESAMT [17] within the CCP4i [18] graphical user interface and displayed with Pymol. C) amino acid sequence alignment of PTBP1.4 and PTBP2. Dots and dashes indicate residues that are identical between the two proteins and regions that are missing in PTBP2 respectively. Residues that are part of the nuclear localization sequence are indicated as NLS. Vertical lines above the sequence indicate residues that make contact with the bound RNA substrate [16]. Pink rectangles indicate regions that interact with the Raver 1 protein [19,20].

PTBP1 represses the inclusion of many neuron-specific regulated exons such as the *c-Src N1* exon, but PTBP2 does not [3,5,7,8]. This difference in splicing regulation plays a critical role in the process of neuronal differentiation and maturation; neuronal progenitor cells express PTBP1 however, during differentiation the level of PTBP1 goes down, while that of PTBP2 goes up [9,10]. PTBP1 expression is repressed, at least in part, due to the action of micro-RNA miR124 [11,12]. This change in protein concentration alters the splicing of a set of neuronal exons that are sensitive to PTBP1 and are critical for the development of axons, dendrites and the formation of synapses. Later, during neuronal maturation, the level of PTBP2 also reduces. This in turn leads to a change in the splicing patterns of many transcripts required for neuronal maturation and adult brain development. Thus, changes in the level of expression of the PTB proteins coupled with their ability to distinctly regulate certain exons plays an important role in the process of neuronal development and maturation [10,13]. PTBP1 can compensate for mice lacking PTBP2 in some developmental contexts but not all; PTBP2 is necessary for neuronal development and maturation in the cerebellum, striatus and brain stem [14]. These studies signify the paralogs have distinct tissue-specific functions. Yet how related proteins that share 74% sequence identity, similar domain organization, near identical RNA interacting residues, recognize and bind to identical *cis* elements in vivo achieve this difference in function is not well understood [1,14–16] (Fig 1).

Reports to date highlight some differences between the two proteins; PTBP2 binds to the sequence downstream of the N1 exon with higher affinity than PTBP1 [5]. PTBP2 also assembles a protein complex in the downstream region that enhances splicing of the N1 exon but PTBP1 does so to a much lesser extent [5]. PTBP1 interacts with the protein Raver 1 more strongly than PTBP2 albeit sharing a near identical interacting motif (Fig 1C) [21] and the paralogs have distinct protein-protein interactions under splicing conditions containing HeLa nuclear extract [22]. Collectively, these reports signify the paralogs have different RNA binding affinities and protein-protein interactions suggesting additional features such as chemical modifications (that might be distinct between the paralogs) might play a role in mediating these differences.

In neuronal WERI nuclear extract, PTBP1 represses splicing of the N1 exon but PTBP2 does so to a much lesser extent [5]. Under these conditions, PTBP1 binds to CUCUCU elements upstream and downstream of the N1 exon to prevent its inclusion in the spliced mRNA. Under the same conditions, PTBP2 does not bind to the upstream region in the presence of ATP likely leading to the observed lesser extent in N1 exon splicing repression [8]. Moreover, the N1 exon is spliced into the final mRNA in neuronal LAN5 cells which do not express PTBP2 [5]. Together, these findings indicate that PTBP2 does not play a role in N1 exon splicing. Rather, its RNA binding affinity and/or protein-protein interactions are modulated differently to that of PTBP1 in neuronal extract. Thus, the WERI retinoblastoma neuronal extract contains factors that can modulate the paralogs’ ability to target distinct exons. To gain a better understanding of these factors, in this study, we aimed to determine 1) protein-protein interactions and 2) chemical modifications that occur in the paralogs in splicing reaction mixtures containing WERI retinoblastoma nuclear extract.

## Experimental Procedures

### Purification of His-tagged PTB proteins from splicing reaction mixtures for mass spectrometry analysis for protein-protein interactions

His-tagged PTBP1 and PTBP2 were prepared as previously described via recombinant protein expression in *E.coli* BL21(DE3) cells and protein expression induced by IPTG [23]. Recombinant proteins were purified using Ni^2+^-affinity chromatography as described previously.

WERI nuclear extract was prepared as previously described [23,24]. Splicing reactions contained 2.2 mM MgCl_2_, 0.4 mM ATP, 20 mM creatine phosphate, 48 ug of histidine tagged PTBP, and were brought to a total volume of 200 µl with WERI retinoblastoma nuclear extract (PTBP1 WERI, PTBP2 WERI) or with Buffer DG (20 mM HEPES KOH pH 7.9, 20% glycerol, 80 mM KCl, 0.2 mM PMSF, 1 mM DTT) for controls (PTBP1 DG, PTBP2 DG). A splicing reaction that contained all components including WERI extract but not PTB protein was also used as a control to identify non-specific proteins inherent to WERI nuclear extract that may stick to the beads (WERI only). Reaction tubes were incubated at 30^o^C for 90 minutes. All steps described here in were carried out at 4^o^C. After incubation,100 µl HisPur Ni-NTA magnetic resin (Thermo Fisher), equilibrated in Buffer DG with 1X Phosstop (Sigma Aldrich) and 1X Sigmafast EDTA free (Sigma Aldrich), was added to each reaction mixture. An additional 200 µl of Buffer DG with 1X Phosstop and 1X Sigmafast was added to each sample and set to incubate overnight. A magnetic separation rack was utilized to remove the buffer. Resin was washed once with 1 ml of Wash Buffer (50 mM sodium phosphate pH 8.0, 150 mM NaCl, 30 mM imidazole, 1X Phosstop, 1X Sigmafast EDTA free) followed by three 1 ml washes with Wash Buffer without 1X Phosstop and 1X Sigmafast EDTA free. 60 µl of Elution Buffer (50 mM sodium phosphate pH 8.0, 150 mM NaCl, 300 mM imidazole) was utilized to resuspend the resin and the samples were set to elute overnight. Supernatant was retrieved via magnetic separation and was dialyzed utilizing Slide-a-lyzer mini units (2000 MWCO) for 2 hours in 1 L of dialysis buffer (20 mM HEPES KOH pH 7.9, 80 mM KCl, 1mM DTT, 0.1 mM PMSF), changing to fresh buffer at the 1-hour mark. To determine PTBP specific protein-protein associations that occur under splicing conditions, eluates equivalent to 5 µg of protein were aliquoted and sent for mass spectrometry analysis to the University of California San Diego, Biomolecular and Proteomics Mass Spectrometry facility.

### SDS-PAGE

Dialyzed elution fractions (7 ul) were prepared for electrophoresis by adding Laemmli SDS-gel loading dye (Bioland Scientific) and heating on a heat block at 95°C for 2 minutes. Samples were loaded into a NuPAGE 4–12% Bis-Tris gel (Thermo Scientific) for gel electrophoresis at 60V for 45 minutes followed by 120V for 1 hour 30 minutes. The gel was stained using Coomassie Blue stain.

### Mass spectrometry analysis of protein-protein interactions

PEAKS 8.5 software was utilized by the mass spectroscopy facility to export the data into an Excel format. For each sample only proteins with an “Area” greater than 0 were utilized. To determine unique proteins found in each PTBP WERI sample, the proteins found in the WERI only control and the DG samples were removed from their respective PTBP1 WERI or PTBP2 WERI list using Excel matching functions. Cytoskeletal proteins (actin, myosin, and tubulin) were removed from this list via Gene Ontology Biological Process annotations to produce a usable list of unique proteins found in PTBP1 WERI and PTBP2 WERI samples respectively. The proteins were identified using their gene name, and the protein lists were then uploaded into the Gene Consortium Gene Ontology website to compare similarities and differences between the types of proteins found in each sample. This process was repeated in order to determine the common proteins found in both PTBP1 WERI and PTBP2 WERI samples. Heatmapper.ca was utilized to produce a heat map to compare the “Area” values of the common PTBP WERI proteins.

## Gene Ontology Analysis

To identify biological processes and molecular functions, the gene names of the proteins were entered into the Gene Consortium Gene Ontology website, and the PANTHER GO-Slim Biological Process and PANTHER GO-Slim Molecular Function were used respectively. Log graphs of observed versus expected were produced to determine the processes and functions these proteins are involved in. Higher values on the graph signify higher occurrence of processes or functions were observed and/or the less they were expected according to algorithms used by Gene Ontology.

### Purification of His-tagged PTB proteins from splicing reaction mixtures for mass spectrometry analysis of post-translational modifications

His-tagged PTBP1 and PTBP2 were prepared as previously described via recombinant protein expression in *E.coli* BL21(DE3) cells and protein expression induced by IPTG [23]. Recombinant proteins were purified using Ni^2+^-affinity chromatography as described previously.

Purified His-tagged PTBP1 or PTBP2 (12 µg) was incubated in splicing conditions (2.2mM MgCl_2_, 0.4mM ATP, 20mM creatine phosphate, brought to 50µl with WERI retinoblastoma nuclear extract) at 30°C for 90 minutes. All subsequent steps were conducted at 4°C. 20µl of HisPur Ni-NTA magnetic resin (Thermo Fisher) was equilibrated in buffer Dialysis Glutamate (Buffer DG) with inhibitors [20mM Hepes-KOH (pH 7.9), 80mM KCl, 0.2mM PMSF, 1mM DTT, 20% glycerol, 3X Phosstop (Sigma Aldrich), 1X Sigmafast EDTA free (Sigma Aldrich), 2µM Vorinostat (MedChemExpress), 5µM Entinostat (MedChemExpress)] then added to splicing reaction and 50µl of buffer DG with inhibitors. Reaction mixtures were incubated overnight at 4°C with agitation at 30 rpm. The resin was washed thrice with 1 ml wash buffer (50mM sodium phosphate (pH 8.0), 150mM NaCl, 30mM imidazole, 1X Phosstop (Sigma Aldrich), 1X Sigmafast EDTA free (Sigma Aldrich), 2µM Vorinostat, 5µM Entinostat (MedChemExpress). Proteins were set to elute overnight with 25µl elution buffer [(50mM sodium phosphate (pH 8.0), 150mM NaCl, 300mM imidazole, 1X Phosstop (Sigma Aldrich), 1X Sigmafast EDTA free (Sigma Aldrich), 2µM Vorinostat (MedChemExpress), 5µM Entinostat (MedChemExpress)] with agitation. The eluates were recovered using a magnetic separation rack and separated on a NuPAGE gel via electrophoresis. The gel band corresponding to the molecular weight of either PTBP1 or PTBP2 were cut out using scalpels, diced into 1 mm cubes, and transferred to 1.5 mL microcentrifuge tubes following sample preparation guidelines provided by the University of California at San Diego (UCSD) Biomolecular and Proteomics Mass Spectrometry Facility (BPMS). The gel pieces were washed and stored in Nanopure water and shipped to the UCSD BPMS facility.

### SDS-PAGE

The eluates (10 ul) were prepared for electrophoresis by adding Laemmli SDS-gel loading dye (Bioland Scientific) and heating on a heat block at 95°C for 2 minutes. Samples were loaded onto a NuPAGE 4–12% Bis-Tris gel (Thermo Scientific) for gel electrophoresis at 60V for 45 minutes followed by 120V for 1 hour 30 minutes. The gel was stained with GelCode Blue Safe Protein stain (Thermo Scientific).

## Mass spectrometry related methods

### In solution sample digestion

**~10 ug of protein sample was** diluted in TNE (50 mM Tris pH 8.0, 100 mM NaCl, 1 mM EDTA) buffer. RapiGest SF reagent (Waters Corp.) was added to the mix to a final concentration of 0.1% and samples were boiled for 5 min. TCEP (Tris (2-carboxyethyl) phosphine) was added to 1 mM (final concentration) and the samples were incubated at 37°C for 30 min. Subsequently, the samples were carboxymethylated with 0.5 mg/ml of iodoacetamide for 30 min at 37°C followed by neutralization with 2 mM TCEP (final concentration). Proteins samples prepared as above were digested with trypsin (trypsin:protein ratio – 1:50) overnight at 37°C. RapiGest was degraded and removed by treating the samples with 250 mM HCl at 37°C for 1 h followed by centrifugation at 14000 rpm for 30 min at 4°C. The soluble fraction was then added to a new tube and the peptides were extracted and desalted using C18 desalting columns (Thermo Scientific, PI-87782). 10% of dissolved peptides was injected for LC-MS analysis.

### LC-MS/MS analysis

#### LC-MS-MS.

Trypsin-digested peptides were analyzed by ultra high pressure liquid chromatography (UPLC) coupled with tandem mass spectroscopy (LC-MS/MS) using nano-spray ionization. The nanospray ionization experiments were performed using a Orbitrap fusion Lumos hybrid mass spectrometer (Thermo) interfaced with nano-scale reversed-phase UPLC (Thermo Dionex UltiMate™ 3000 RSLC nano System) using a 25 cm, 75-micron ID glass capillary packed with 1.7-µm C18 (130) BEH^TM^ beads (Waters corporation). Peptides were eluted from the C18 column into the mass spectrometer using a linear gradient (5–80%) of ACN (Acetonitrile) at a flow rate of 375 μl/min for 1.5 h. The buffers used to create the ACN gradient were: Buffer A (98% H_2_O, 2% ACN, 0.1% formic acid) and Buffer B (100% ACN, 0.1% formic acid). Mass spectrometry analysis was carried out using the Bruker TimsTOF Pro 2. For the timsTOF Pro 2 settings, the following parameters were adapted, starting from the PASEF method for standard proteomics. The values for mobility-dependent collision energy ramping were set to 95 eV at an inversed reduced mobility (1/*k*_0_) of 1.6 V s/cm^2^ and 23 eV at 0.73 V s/cm^2^. Collision energies were linearly interpolated between these two 1/*k*_0_ values and kept constant above or below. No merging of TIMS scans was performed. Target intensity per individual PASEF precursor was set to 20 000. The scan range was set between 0.6 and 1.6 V s/cm^2^ with a ramp time of 166 ms. 14 PASEF MS/MS scans were triggered per cycle (2.57 s) with a maximum of seven precursors per mobilogram. Precursor ions in an *m*/*z* range between 100 and 1700 with charge states ≥3+ and ≤8 + were selected for fragmentation. Active exclusion was enabled for 0.4 min (mass width 0.015 Th, 1/*k*_0_ width 0.015 V s/cm^2^). Protein identification and label free quantification was carried out using Peaks StudioPro X (Bioinformatics solutions Inc.).

#### LC-MS-MS.

Trypsin-digested peptides were analyzed by ultra high pressure liquid chromatography (UPLC) coupled with tandem mass spectroscopy (LC-MS/MS) using nano-spray ionization. The nanospray ionization experiments were performed using a Orbitrap fusion Lumos hybrid mass spectrometer (Thermo) interfaced with nano-scale reversed-phase UPLC (Thermo Dionex UltiMate™ 3000 RSLC nano System) using a 25 cm, 75-micron ID glass capillary packed with 1.7-µm C18 (130) BEH^TM^ beads (Waters corporation). Peptides were eluted from the C18 column into the mass spectrometer using a linear gradient (5–80%) of ACN (Acetonitrile) at a flow rate of 375 μl/min for 1.5 h. The buffers used to create the ACN gradient were: Buffer A (98% H_2_O, 2% ACN, 0.1% formic acid) and Buffer B (100% ACN, 0.1% formic acid). Mass spectrometer parameters are as follows; an MS1 survey scan using the orbitrap detector (mass range (m/z): 400–1500 (using quadrupole isolation), 60000 resolution setting, spray voltage of 2400 V, Ion transfer tube temperature of 285 C, AGC target of 400000, and maximum injection time of 50 ms) was followed by data dependent scans (top speed for most intense ions, with charge state set to only include +2–5 ions, and 5 second exclusion time, while selecting ions with minimal intensities of 50000 at in which the collision event was carried out in A- high energy collision cell (HCD Collision Energy of 30%), and the fragment masses where analyzed in the ion trap mass analyzer (With ion trap scan rate of turbo, first mass m/z was 100, AGC Target 5000 and maximum injection time of 35ms), followed by B) and the fragment masses where analyzed in the ion trap mass analyzer (With ion trap scan rate of turbo, first mass m/z was 100, AGC Target 5000 and maximum injection time of 35ms), Data analysis for N- and O linked glycosylation site determination was carried out using the Peaks Studio Pro X (Bioinformatics solutions). For the Thermo Fusion Orbitrap LUMOS runs the: PEAKS Version: PEAKS Studio 10.6 build 20201221.

### Mass spectrometry data access

The mass spectrometry proteomics data have been deposited to the ProteomeXchange Consortium via the PRIDE [25] partner repository with the dataset identifier PXD058740.

## Results

### The paralogs interact with proteins related to pre-mRNA processing and chemical modification “reader” proteins

**Fig 2:**
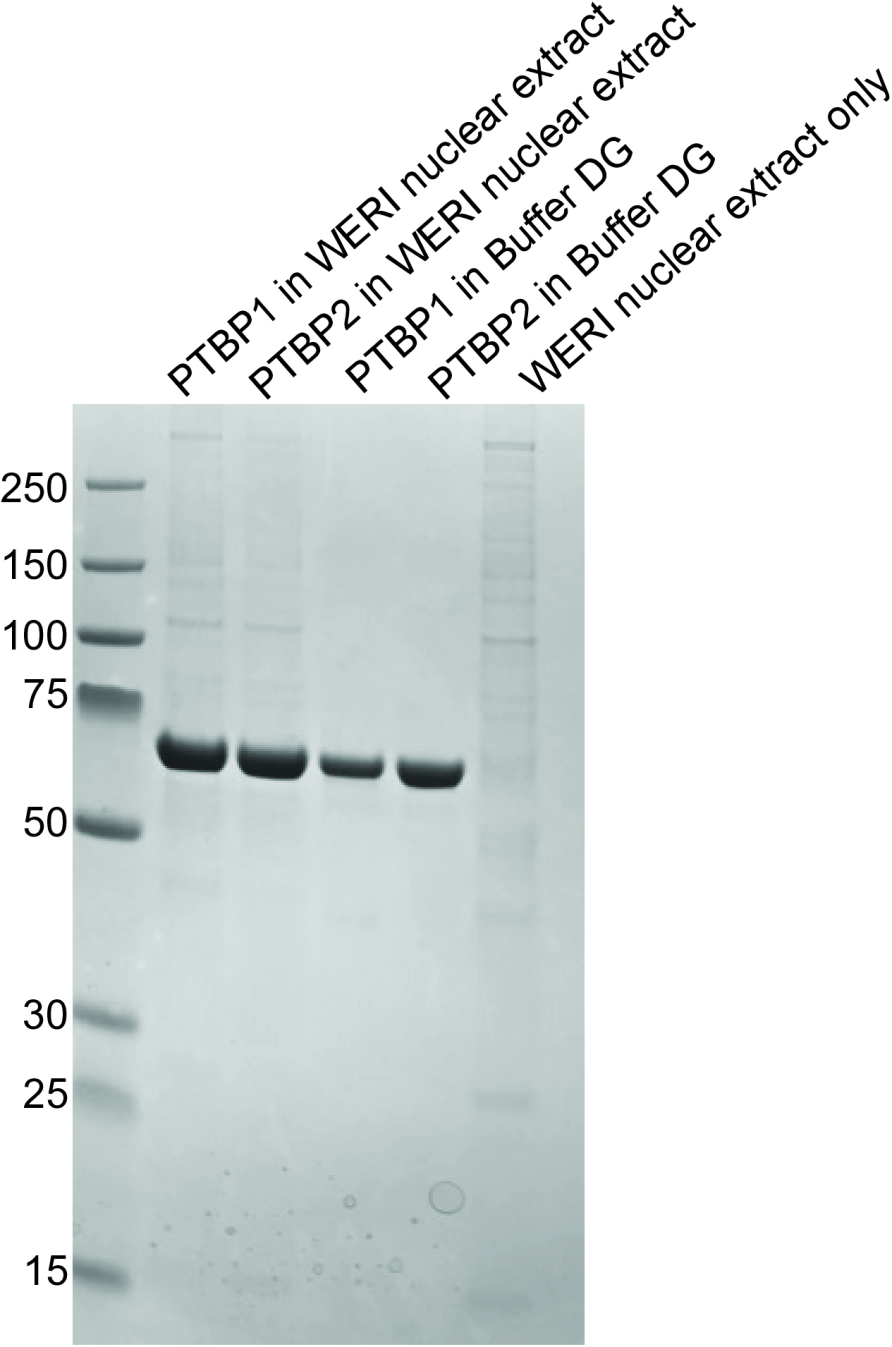
Purification of His-_6_ tagged PTBP from splicing reaction mixtures. Splicing reaction mixtures contained 2.2mM MgCl_2_, 0.4mM ATP and 20mM Creatine Phosphate and either WERI nuclear extract or Buffer DG. Aliquots (5 μl) of the indicated fractions were analyzed by SDS-PAGE. The gel was stained with Gel Code blue safe stain. The positions and sizes (kilodaltons) of marker polypeptides are shown on the left.

To determine proteins associated with PTBP1 and PTBP2 in neuronal cells, we incubated recombinant expressed His-tagged PTB proteins under splicing reaction conditions containing WERI nuclear extract as previously described [22,23]. PTB proteins were pulled down from these reaction mixtures using Ni^2+^ magnetic beads. PTB proteins incubated under splicing conditions containing the dialysis buffer used in extract preparation (Buffer DG) served as controls. A reaction that contained all components of the splicing reaction mixture including WERI nuclear extract, without the addition of PTB proteins served as a control to determine nonspecifically bound proteins present in the eluates. Elution fractions were analyzed for purity and the presence of co-purified proteins via SDS-PAGE Coomassie stain (Fig 2). An intense band at ~57 kDa in all lanes confirms the presence of recombinant PTBP1 and PTBP2 in all elution fractions except the WERI only control.

**Fig 3:**
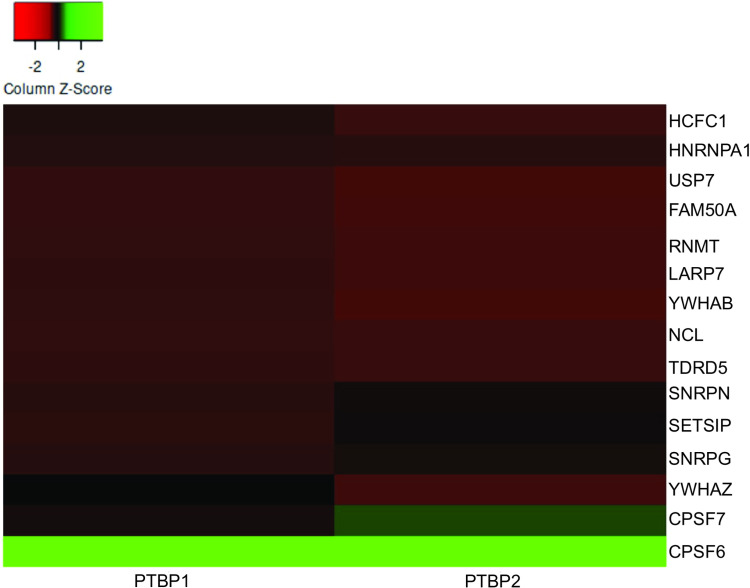
Heat map depicting the relative prevalence of the common proteins pulled down with both PTBP1 and PTBP2. The 0 baseline, indicated by black color is calculated by taking the average area score of all the common proteins. Proteins with abundance higher or lower than the average are indicated by green or red color respectively. Each point on the scale shows one standard deviation from the average. Heatmap was generated using Heatmapper.ca.

We observe the presence of many proteins in the WERI only control indicative of nonspecifically bound proteins. We analyzed the protein composition of each of the eluates via mass spectrometry. To determine proteins specific and unique to the paralogs in neuronal nuclear extract we removed proteins present in the PTBP1 DG sample (representative of proteins that came down during PTBP1 purification from bacterial cells) (S1 Table), PTBP2 DG sample (representative of proteins that came down during PTBP2 purification from bacterial cells) (S2 Table) and the WERI only control (representative of proteins in the WERI extract that stuck nonspecifically to the beads) (S3 Table) from the PTBP1 WERI and PTBP2 WERI lists. We also removed commonly occurring contaminants including keratin, cytoskeletal proteins and those described in previous reports [26]. We next compared the lists of proteins for the paralogs and identified 15 proteins present in both samples (S4 Table) that were deemed common associated proteins. A heatmap was created to visually compare the abundance of each protein in PTBP1 vs PTBP2 eluate (Fig 3). We note the paralogs interacted with proteins involved in pre-mRNA processing, transcription and chemical modifications. The nuclear extract used in the assay includes RNA transcripts. Thus, proteins present in the eluates may either be directly interacting with the PTB proteins or are bound to RNA transcripts that are simultaneously bound by the His-tagged PTB protein. Our data indicate the cleavage and polyadenylation factor 6 (CPSF6) interacted with both proteins with the highest abundance (Fig 3). CPSF6 is required for 3’ RNA cleavage and polyadenylation processing. Both PTBPs co-purified with CPSF7, a component also involved in 3’ RNA cleavage and polyadenylation. Given the interdependence of splicing with cleavage and poly A tail addition, the presence of these components on RNA transcripts is not surprising [27]. PTB proteins also interact with small nuclear ribonuclear proteins SNRNPN, SNRNPG and HNRNPA1 consistent with their role in regulating alternative splicing. Both proteins co-purified with transcription regulators LARP7, SETSIP, mRNA cap methyltransferase RNMT and with FAM50A which plays a role in mRNA processing via the spliceosome C complex [28]. Given the tight associations between proteins involved in transcription, capping, splicing, cleavage and polyadenylation, it is likely that co-purified proteins are associated with gene transcripts undergoing processing [27]. PTBP1 and PTBP2 copurified with the Tudor- domain containing protein TDRD5. Tudor domain containing proteins bind to methylated arginine and lysine residues [29]. This result supports the presence of post translational methylation at lysine and arginine residues in the PTB proteins and/or association of PTBPs with proteins that have these modifications. The paralogs interact with YWHAZ and YWHAB which belong to the 14-3-3 family of proteins that mediate cellular signaling by binding to phosphoserine containing proteins. Given that PTBP1 and PTBP2 are phosphorylated [23,30] it is plausible that either YWHAZ/B are binding via recognition of these phosphorylated residues, or they are interacting with other phosphorylated proteins bound to RNA transcripts. The paralogs interact with deubiquitinating enzyme USP7 that can play a role in removing ubiquitin and protecting the PTBPs from degradation. This interaction and other evidence suggest the PTB proteins are ubiquitinated [30]. Overall, the list of common proteins is aligned with known functions of the paralogs and also support the presence of chemical modifications on the PTB proteins.

### PTBP2 is associated with proteins involved in splicing, chromatin remodeling and chemical modifications

**Fig 4:**
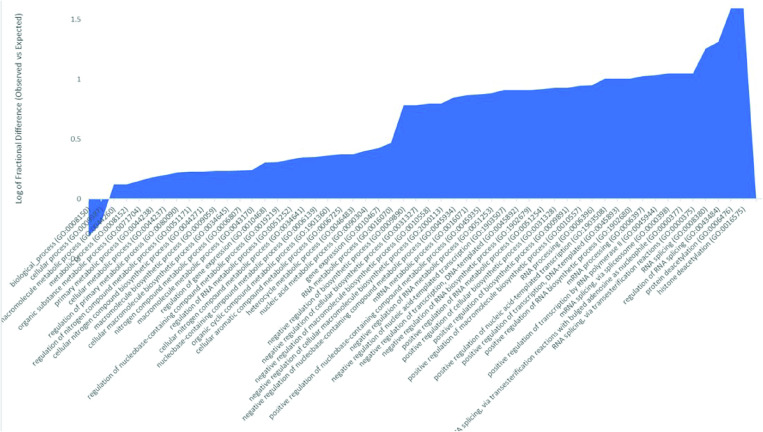
Log fractional difference of observed vs expected PANTHER GO-Slim biological process categories assigned to proteins found unique to the PTBP2 pulldown. The log fractional difference is calculated for each category as (# genes for the category-# genes expected)/ # genes expected) as provided on Gene Ontology. Highest processes on this graph include histone and macromolecule deacetylation, regulation of RNA splicing, and transcription regulation.

Here we analyzed proteins that were present and unique to the PTBP2 pull down eluate. We identified a list of 39 unique proteins that interacted either directly with PTBP2 or with mRNA transcripts bound by PTBP2 (S5 Table). Gene Ontology analysis of the biological function of these co-purified proteins reveals they participate in several processes including mRNA processing, splicing and transcription regulation (Fig 4). We observed that a given protein could be in one or more of these categories. A positive value of the log of fractional difference between observed vs expected indicates a higher occurrence of genes (of corresponding proteins) with the indicated function compared to the expected number. Thus, we note the highest fold difference is for protein deacetylation (Fig 4) followed by splicing and regulation of transcription. Gene ontology analysis of the molecular function of proteins unique to the PTBP2 eluate align with functions described above including transcription co-repression, and transcription factor, chromatin and RNA binding activity (S1 Fig).

To gain a better understanding about the kinds of processes PTBP2 might be participating in neuronal cells (and if they might provide insight into its ability to regulate distinct sets of exons) we analyzed the proteins that co-eluted in detail. To this end, PTBP2 interacts with serine arginine rich splicing factors SRSF1, SRSF11, SRSF8, U4/U6. U5 tri snRNP associated proteins SNRNP27, USP39, and heterogenous ribonuclear proteins hnRNPH1and hnRNPU consistent with its role in regulating alternative pre-mRNA splicing. The presence of MAGOH and MAGOHB, components of the exon junction complex (EJC) suggest the association of PTBP2 directly with the EJC or with mRNA transcripts that are being actively spliced. We note the presence of elongation factor EEF1A1 that can enter the nucleus and directly bind to cellular RNAs at the 3’ end and provide stability [31]. We observe the presence of Matrin 3 that has a known PTBP interacting peptide motif in RNA binding domain 2 (Fig 1C). Thus, although both paralogs have identical interacting motifs PTBP2 but not PTBP1 interacts with Matrin 3 in neuronal nuclear extract under the assayed conditions [32]. PTBP2 interacts with ANP32B, an acidic leucine rich phosphoprotein that is thought to have a role in neuronal differentiation [33]. Our results highlight that PTBP2 interacts with both hnRNPA1 and DDX5 (a DEAD box helicase involved in multiple aspects of RNA processing). These two proteins work coordinatively to regulate alternative splicing of pre-mRNA targets [34]. PTBP2 also interacts with proteins SKP1 and ZSWIM8, components of the E3 ubiquitin protein ligase family [35,36].

We found histone lysine demethylase RIOX2 in the eluates with PTBP2. We surmise RIOX2 could be interacting with histones and copurified with PTBP2 in a large active transcription complex or that RIOX2 interacts with PTBP2. The latter suggests PTBP2 may be modified via methylation. Demethylation can be associated with euchromatin and active transcription. To this end, PTBP2 interacts with TBPL1, a TATA box-binding protein like −1 that plays an important role in transcription by RNA Polymerase II as components of the transcription factor IID complex [37]. Enzymes peptidyl-prolyl cis-transisomerase (PPIL3), DNA methyltransferase (DNMT1), and serine threonine protein kinase like protein PXK are enzymes that co-purified with PTBP2 via either direct or indirect association. These findings support the presence of PTM’s in PTBP2 in neuronal cells. Proteins MTA1 and MTA3 that are part of the NuRD chromatin remodeling complex co-purified with PTBP2 [38]. The deacetylase subcomplex of NuRD consists of MTA1, MTA2 and/or MTA3 and HDAC1/2 and histone chaperone proteins RBBP4/7. De-methylated MTA1 associates with a remodeling complex that is actively engaged in gene transcription [39]. PTBP2 also co- purified with the SWI/SNF chromatin remodeling complex components SMARCB1, SMARCE1, SMARCA2 and SMARCC2 indicating the presence of chromatin and genes activated for transcription [40,41]. Collectively, our data suggest that PTBP2 is bound in part to RNA transcripts being actively transcribed and are still associated to the DNA template. These findings align with previous reports regarding the influence of chromatin on pre-mRNA splicing and transcription enhancement [27,42]. Our data also support the presence of chemical modifications in PTBP2.

### PTBP1 is associated with unique proteins under neuronal splicing conditions

**Fig 5:**
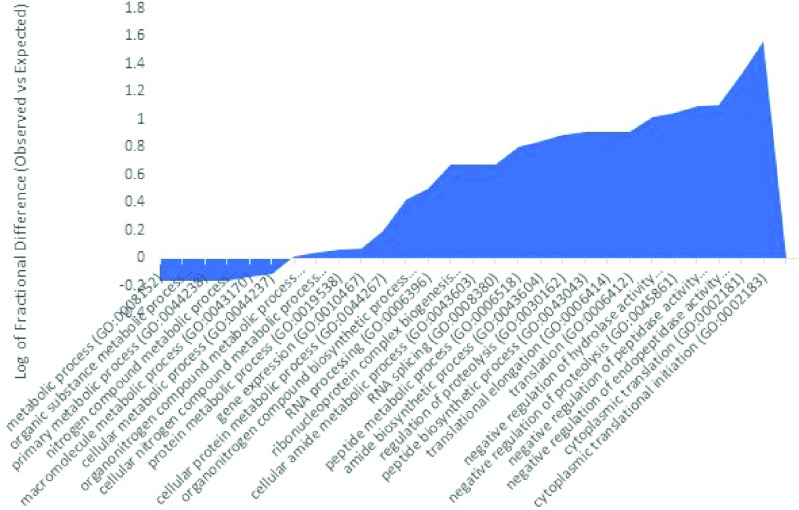
Log fractional difference of observed vs expected PANTHER GO-Slim biological process categories assigned to proteins found unique to the PTBP1 pulldown. The log fractional difference is calculated for each category as (# genes for the category-# genes expected)/ # genes expected) as provided on Gene Ontology. Highest processes on this graph are related to the regulation of cytoplasmic translation.

Our results indicate that PTBP1 interacted with 112 unique proteins (S6 Table). Gene Ontology (GO) analysis of the biological function of these proteins revealed the top functions to be related to translation and ribosome structure followed by RNA processing and splicing at a value more than 2-fold lower (Fig 5). Gene Ontology analysis of the molecular function of these proteins align well with the biological functions (S2 Fig). As indicated in S6 Table, the PTBP1 pull-down eluate included many ribosomal proteins RPSA, RPS2, RPS3, RPS11, RPS16, RPS20, RPS25, RPLP1, RPLP2, RPL3, RPL7, RPL8, RPL11, RPL12, RPL13A, RPL18, RPL19, methyltransferase EMG1, translation initiation factors EIF4A1, EIF4A2, and elongation factors EEF1D, EEF1B2 and EIF2S3. To date, there are no reports that describe either the association of hnRNPs including PTBP1 to rRNA or the association of ribosomal proteins to nuclear Polymerase II RNA transcripts. Thus, these scenarios fail to explain the presence of ribosomal proteins with PTBP1 incubated in neuronal nuclear extract. PTBP1 shuttles between the nucleus and cytoplasm to carry out its roles in the two compartments [43,44]. In the cytoplasm, PTBP1 participates in internal ribosome entry site (IRES) mediated translation initiation of both viral and cellular RNAs [45–47]. PTBP1 export from the nucleus to the cytoplasm is mediated by an export sequence in the N-terminal region and RNA binding domain 2 (RRM2) [43,48]. PTBP1 export is not mediated by the transportin family member Crm1 and the identity of the cellular receptor that mediates PTBP1 export is unknown [48]. At steady state, PTBP1 is primarily nuclear. Studies highlight that PTBP1 export is not mediated by RNA export but rather by protein-protein interactions [43]. Our results support a direct PTBP1-ribosomal protein/translational initiation factor association. Moreover, PTBP1 RRM2 region interacts with ribosomal proteins RPS 25, RPS 26, RPS 27, RPL12, RPL 22, EIF1A (NK unpublished data). Ribosome subunit export is dependent on Ran proteins, and we observe the presence of Ran-related protein RCC1 in the list of PTBP1 co-purified proteins (S6 Table). Moreover, ribosome subunit export is energy dependent like PTBP1 [43,49]. Collectively, our findings suggest that PTBP1 export is mediated by interaction with ribosomal proteins via the RRM2 region. This notion will be tested in future work in the laboratory. It is noteworthy that ribosomal proteins didn’t co-purify with PTBP2. Thus, contacts mediating PTBP-ribosomal protein interactions appear to be distinct between the paralogs under the assayed conditions.

PTBP1 interacted with a number of H2A variant proteins (S6 Table). We found splicing factors snRNPA, SF3B2, U2AF2, hnRNPD, hnRNPLL, hnRNPK, and DDX39B that co-purified with PTBP1 and is related to its role in alternative splicing regulation [50]. For exons repressed by PTBP1, spliceosome assembly is blocked at the A Complex [51,52]. To this end, we note the absence of pre-catalytic spliceosome B Complex proteins including U4/U6.U5 tri-snRNP associated proteins that co-purified with PTBP2. PTBP1 also co-eluted with CSTF2T involved in pre-mRNA 3’ end processing. We note the presence of CHD5, a protein involved in chromatin remodeling, proteins involved in transcriptional regulation and splicing including MEIS2, PUF60, NONO, FUBP1 and TAF8 that is part of the TFIID basal transcription factor complex. We note the presence of transcriptional regulatory factors TRIM28 and TRIM29 in the list of proteins unique to PTBP1. It is plausible these may either interact directly with PTBP1 or were pulled down indirectly via interaction with the histone proteins.

Our results highlight the presence of several proteins involved in cell signaling that co-eluted with PTBP1. These include the 14-3-3 adaptor protein SFN that recognizes phosphoserine and phosphothreonine residues, CALM1 and CALM2 involved in calcium-sensing cell signaling, Ras-related proteins RAB11A, RAB7A, RAB2A, GDI2, the WW-domain containing proteins involved in cell signaling such as WAC. We note the presence of PABPC1 in the list of proteins unique to PTBP1. PABPC1 plays a role in mRNA stability and translation initiation [53]. PTBP1 co-purified with PRRC2A, a m6A modification reader in oligodendrocytes indicating direct interaction with PRRC2A or with m6A modified transcripts.

Finally, we observe proteins related to the ubiquitin/19S regulatory subunit of the proteasome complex, TBL1X, TBL1Y, PSMD12, a protein related to the 20S core proteasome complex PSMA6 and an E3 ubiquitin ligase LTN1 that promotes degradation. Collectively, analysis of the list of proteins that co-eluted with PTBP1 highlight the presence of proteins related to well-characterized PTBP1 functions including splicing regulation. It is noteworthy that our findings support a role for ribosomal proteins as candidates that mediate PTBP1 nuclear export.

### PTBP1 and PTBP2 are post-translationally phosphorylated in neuronal nuclear extract

**Fig 6:**
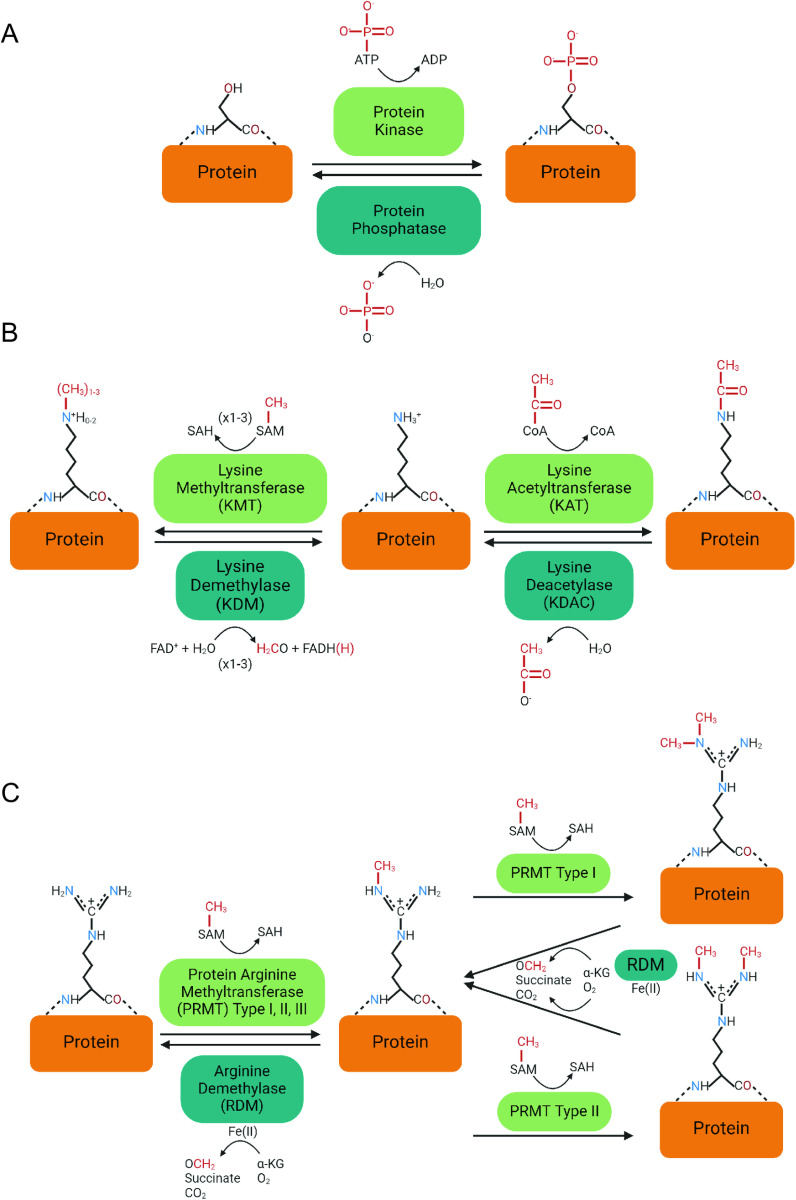
Enzymatic reactions for reversible covalent modifications. (A) protein kinase catalyzes the phosphorylation of serine, using ATP as a cofactor in the reaction. A protein phosphatase catalyzes the removal of the phosphate group via hydrolysis releasing an inorganic phosphate. (B right) lysine acetyltransferase (KAT) catalyzes the addition of an acetyl group using acetyl-CoA as a cofactor. A lysine deacetylase (KDAC) catalyzes the removal and release of the acetyl group. (B left) lysine methyltransferase (KMT) uses *S*-adenosyl methionine (SAM) as a methyl donor to add a methyl group to the epsilon amino group of a lysine side chain. SAM is converted to *S*-adenosyl homocysteine (SAH) during the reaction. The lysine can be mono-, di-, or tri-methylated by this mechanism. A lysine demethylase (KDM) will oxidize the methyl group using oxidized Flavin Adenine Dinucleotide (FAD^+^) as a cofactor and release formaldehyde. **(C)** A guanidino group of arginine side chains is monomethylated by a protein arginine methyltransferase (PRMT), which can occur by type I, II, or III using a SAM cofactor [55,56]. Type I PRMT can further methylate the arginine, making asymmetric di-methyl-arginine (ADMA), while type II makes symmetric di-methyl-arginine (SDMA). Demethylation occurs by an arginine demethylase (RDM) catalyzing the oxidative decarboxylation of α-ketoglutarate (α-KG) to form succinate, carbon dioxide, and an iron **(IV)**-oxo intermediate, further reacting and releasing formaldehyde [55,56].

Analysis of protein-protein interactions of PTBP1 and PTBP2 under neuronal nuclear extract highlight the paralogs have distinct protein-protein interactions albeit high structural similarity. Thus, we hypothesized factors on top of the amino acid sequence such as reversible covalent modifications alter their chemical composition and in turn protein-protein interactions. To this end, we conducted mass spectrometry experiments to identify chemical modifications that occur in PTB proteins when incubated in neuronal WERI nuclear extract. Our results highlight the PTB proteins are modified by the addition of phosphate, acetyl and methyl groups (Tables 1 and 2). Reversible modification via phosphorylation is catalyzed by protein kinases and phosphatases (Fig 6A). For PTBP2, we observe many phosphate modifications in the unstructured N-terminal, Linker 1 and Linker 2 regions as previously described [23]. It is noteworthy that under WERI nuclear extract we also observe phosphate modifications in Linker 3 and the RRM regions (Tables 1 and 2). Phosphate modifications in PTB proteins have been previously described albeit under non-neuronal nuclear extract conditions [23]. We observe high overlap between the two data sets (neuronal and non-neuronal) for PTBP2 phosphorylation in the N-terminal, Linker 1 and Linker 2 region (Table 2). PTBP1 is also phosphorylated under neuronal nuclear extract (Table 1). However, we see a significant difference in the extent of phosphorylation in the N-terminal region between the paralogs where PTBP1 has only one phosphorylated residue and PTBP2 has nine. Molecular dynamics studies indicate that PTBP2 N-terminal region shows significant differences in N/C-termini distance, radius of gyration, partial molar volume, and secondary structure when phosphorylated [54]. We observe phosphate modifications in RRM regions for the paralogs as well, under WERI nuclear extract conditions. PTBP2 unstructured regions play a role in regulating its splicing activity on certain exons including the neuronal N1 exon [54]. Whether phosphorylation in these regions including the N-terminal contributes to PTBP2 splicing activity is currently under investigation in our laboratory.

**Table 1:** Post-translational modifications in PTBP1 under splicing conditions with WERI retinoblastoma nuclear extract.

Domain	Residue No.	% acetylation	% phosphorylation	% monomethylation
N-Terminal Region	T25	---	0.86 ± 0.63	---
	K45	0.42 ± 0.30	---	1.02 ± 0.73
RRM1	K65	4.97 ± 2.75	---	4.92 ± 2.49
	T71	---	8.80 ± 8.25	---
	K84	2.89 ± 1.57	---	3.51 ± 2.22
	K92	2.54 ± 1.23	---	1.54 ± 1.17
	**K94**	3.59 ± 2.26	---	2.09 ± 1.38
	T109	---	0.70 ± 0.51	---
	R122	---	---	0.64 ± 0.42
	K134	2.38 ± 1.19	---	11.51 ± 5.75
	**K137**	4.46 ± 3.18	---	5.40 ± 2.59
	T138	---	8.97 ± 8.23	---
Linker-1	S140	---	2.63 ± 1.69	---
	S156	---	13.99 ± 9.01	---
	S159	---	14.12 ± 8.97	---
	S167	---	11.61 ± 11.01	---
	S181	---	0.36 ± 0.24	---
RRM2	**R185**	---	---	6.73 ± 3.74
	K206	---	---	4.17 ± 2.64
	K212	2.54 ± 1.72	---	---
	T215	---	1.63 ± 1.04	---
	**K218**	1.79 ± 1.53	---	3.04 ± 2.06
	Y228	---	5.78 ± 5.51	---
	K238	1.18 ± 0.55	---	6.21 ± 2.82
	S240	---	17.46 ± 10.32	---
	R254	---	---	16.15 ± 7.73
	**K259**	5.71 ± 3.69	---	---
	**K266**	4.25 ± 2.11	---	---
	R277	---	---	6.22 ± 3.42
Linker-2	S285	---	3.37 ± 2.22	---
	S288	---	1.11 ± 0.73	---
	R351	---	---	3.44 ± 1.54
RRM3	S363	---	0.53 ± 0.42	---
	S368	---	12.50 ± 8.54	---
	R374	---	---	10.20 ± 3.91
	R392	---	---	3.14 ± 2.35
	S418	---	0.63 ± 0.37	---
	K424	---	---	3.98 ± 2.34
	K436	11.36 ± 5.34	---	25.28 ± 15.53
	T453	---	1.40 ± 0.95	---
	K454	6.95 ± 2.34	---	11.37 ± 5.17
	Y456	---	2.52 ± 1.93	---
	S459	---	1.20 ± 0.76	---
Linker-3	R463	---	---	4.83 ± 2.55
RRM4	K497	2.90 ± 1.71	---	13.39 ± 3.51
	K508	3.89 ± 1.90	---	7.04 ± 3.41
	S526	---	1.13 ± 0.92	---

**Table 2:** Post-translational modifications in PTBP2 under splicing conditions with WERI nuclear extract.

Domain	Residue No.	% acetylation	% phosphorylation	% monomethylation	% dimethylation
N-terminal region	R14	---	---	---	1.12 ± 0.49
	S16	---	0.85 ± 0.32	---	---
	S21	---	2.40 ± 1.26	---	---
	S23	---	2.17 ± 0.45	---	---
	S26	---	2.39 ± 0.14	---	---
	S27	---	4.01 ± 0.03	---	---
	S30	---	2.88 ± 0.15	---	---
	S33	---	0.77 ± 0.22	---	---
	S34	---	1.08 ± 0.58	---	---
	S44	---	0.78 ± 0.22	---	---
RRM1	**K94**	---	---	15.87 ± 5.34	---
	T103	---	6.69 ± 0.81	---	---
	T109	---	7.62 ± 1.90	---	---
	R122	---	---	8.23 ± 6.59	---
	K134	3.08 ± 0.14	---	6.31 ± 3.37	---
	**K137**	4.12 ± 0.42	---	---	---
	T138	---	4.55 ± 0	---	---
	T141	---	11.36 ± 2.27	---	---
Linker-1	T154	---	5.11 ± 1.78	---	---
	T161	---	2.80 ± 0.23	---	---
	T174	---	2.15 ± 0.88	---	---
RRM2	**R182**	---	---	---	3.18 ± 0.05
	**K215**	18.27 ± 6.73	---	---	---
	K235	---	---	6.34 ± 1.07	---
	Y244	---	7.14 ± 2.38	---	---
	R251	---	---	20.39 ± 4.61	---
	**K268**	---	---	14.25 ± 1.75	---
	S269	---	4.08 ± 0.08	---	---
	R270	---	---	---	9.32 ± 1.21
	T273	---	5.10 ± 2.60	---	---
	R274	---	---	13.75 ± 3.75	12.50 ± 2.50
Linker-2	S279	---	4.76 ± 0	---	---
	K296	---	---	3.38 ± 1.16	2.25 ± 0.03
	T298	---	14.65 ± 3.54	---	---
	S299	---	29.29 ± 7.07	---	---
RRM3	K375	0.72 ± 0.07	---	3.52 ± 0.37	---
	R419	---	---	---	6.02 ± 1.67
	K429	8.57 ± 1.43	---	5.33 ± 1.33	---
	S434	---	6.83 ± 0.58	---	---
Linker-3	S444	---	12.99 ± 4.66	---	---
RRM4	S454	---	4.96 ± 2.18	---	---
	S460	---	3.08 ± 0.37	---	---
	R472	---	---	15.67 ± 12.90	---
	K483	---	---	13.39 ± 0.89	---

Post-translational acetyl, phosphate, and methyl modifications in PTBP1 incubated in splicing reaction mixtures containing WERI retinoblastoma nuclear extract. Data collected from 6 independent trials shows average percent modification with mean error. Percent modification is calculated by dividing the number of peptides that contain a modified residue by the total number of peptides that contain the residue in a trial. Only modifications that occur in more than one trial are reported. Residues in bold are those that participate in H-bond interactions with the bound RNA substrate (less than 3 Å bond distance) in the NMR structure solutions [16].

Post-translational acetyl, phosphate, methyl and dimethyl modifications in PTBP2 incubated in splicing reaction mixtures containing WERI retinoblastoma nuclear extract. Data collected from 2 independent trials shows average percent modification with mean error. Percent modification is calculated by dividing the number of peptides that contain a modified residue by the total number of peptides that contain the residue in a trial. Only modifications that occur in more than one trial are reported. Residues in bold are those that participate in H-bond interactions with the bound RNA substrate (less than 3 Å bond distance) in the NMR structure solutions of counterpart residues in PTBP1 [16].

### The PTBP paralogs are post-translationally acetylated in neuronal nuclear extract

**Fig 7:**
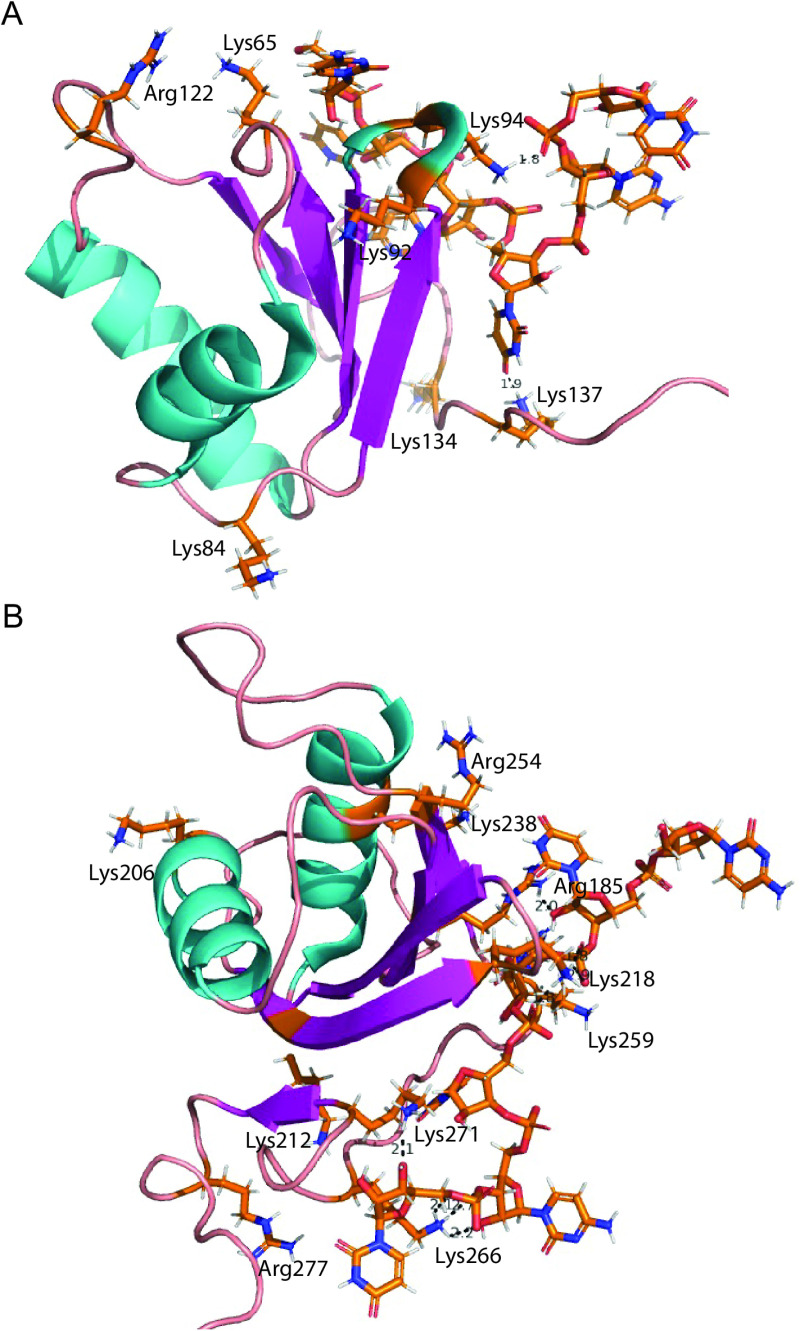
Location and interactions of modified lysine side chains in RRMs 1 and 2. (A) and **(B)** Cartoon representation of NMR solution structures of PTBP1 RRM1 (2AD9) and RRM2 (2ADB) bound to a CUCUCU hexamer (Oberstrass et al. 2005). The main chain cartoon traces are colored by secondary structure. Post-translationally modified residues identified by mass spectrometry are labeled. Side chains of modified residues and the CUCUCU RNA hexamer are shown as sticks and colored by element.

Our results highlight PTBP1 and PTBP2 lysine residues in the RRM regions are modified by reversible acetylation. Acetyl groups are added and removed by lysine acetyltransferases and lysine deacetylases respectively (Fig 6B right). Acetyl modifications are primarily localized to the RNA binding domains for the two paralogs in agreement with published reports [57,58]. PTBP1 is acetylated at many more side chains compared to PTBP2 in the RRM1 region (Tables 1 and 2). We note overlapping modifications between the paralogs at positions Lys134 and Lys137. We used the solution NMR structure of PTBP1 RRM1 bound to a CUCUCU hexamer to determine the positions and interactions of the acetylated lysine side chains (Fig 7A). Lys137 makes an H-bond interaction with the nitrogenous base of U4. Thus, addition of an acetyl group to the **ε**-amino group can disrupt this H-bond interaction, cause steric hinderance and in turn influence sequence recognition and RNA binding affinity. PTBP1 is also acetylated at residues Lys65, Lys84, Lys92 and Lys94. Lys65, 84 and 92 do not make any contact with the bound RNA. Lys65 is near the bound RNA however the **ε-**amino group does not make any H-bond interactions with the substrate. It is noteworthy that rotation around the single C-N bond can position the amino group to form H-bonds with the bound RNA and play a role in binding affinity. Lys94 makes an H-bond interaction with the phosphodiester linkage between C5 and U6 (Fig 7A). Acetylation at this side chain may disrupt this interaction and cause steric hinderance and in turn, play a role in RNA binding affinity. Thus, acetylation in RRM1 may play a role in RNA binding affinity.

We observe a similar pattern for acetylation in RRM2 where PTBP1 is modified at five lysine side chains Lys212, Lys218, Lys238, Lys259 and Lys266 while PTBP2 is acetylated at one position (Lys215) which overlaps with PTBP1 (Lys218). Lys266 makes three H-bond interactions with the phosphodiester backbone between C5-U6 indicating an important role in orienting the substrate and RNA binding affinity. Thus, acetylation at this residue will play a significant role in RNA binding affinity. Similarly, Lys 218 also makes three H-bond interactions with the phosphodiester backbone between C3 and U4 (Fig 7B) and acetylation at this position can also impact RNA binding affinity. Lys212 and Lys238 are pointing away from the RNA substrate. Lys259 is located in close proximity to the substrate and has the potential to interact via H-bond or phi-cation interactions with the C3 nitrogenous base. Thus, our data support a role for acetylation in RRM2 to play a role in RNA binding affinity.

**Fig 8:**
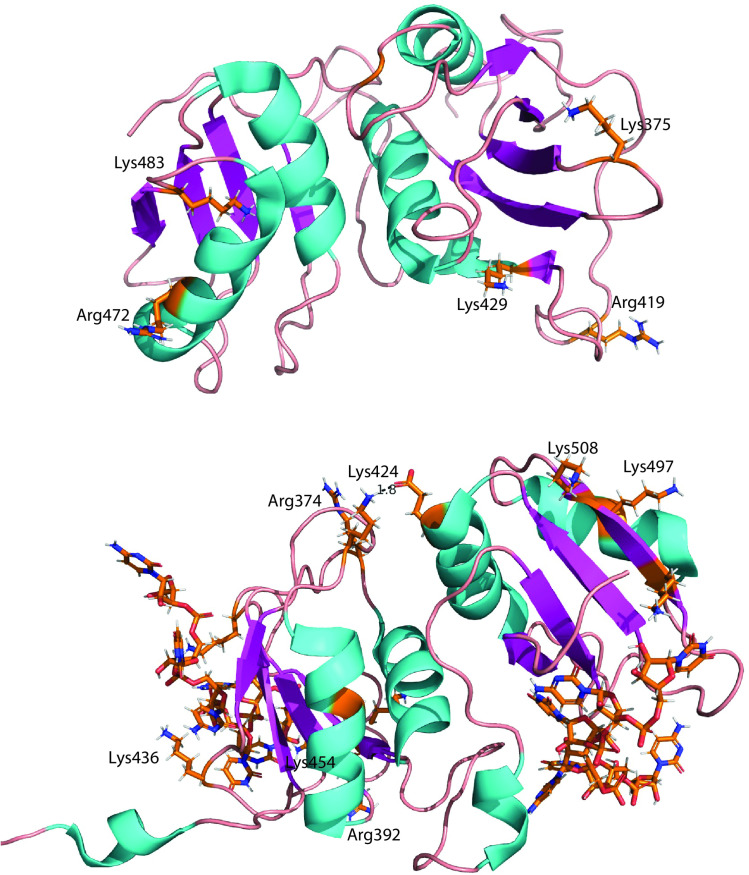
Location and interactions of modified lysine side chains in RRMs 3 and 4. **(A)** and **(B)** Cartoon representation of NMR solution structures of PTBP2 RRM 3_4 (2AD9) and PTBP1 RRM3_4 (2ADB) (Oberstrass et al. 2005 and add Joshi ref). The main chain cartoon traces are colored by secondary structure. Post-translationally modified residues identified by mass spectrometry are labeled. Side chains of modified residues and the CUCUCU RNA hexamer (for PTBP1) are shown as sticks and colored by element.

The paralogs are acetylated at RRM3 at two positions including an over-lapping modification at Lys454 (PTBP1) and Lys429 (PTBP2). PTBP1 is also acetylated at Lys436 (an RNA interacting residue) and PTBP2 at Lys375 (counterpart of PTBP1 Lys 400). Our data reveals the number of acetyl modifications in RRMs3-4 regions is less compared to RRMs 1 and 2 for PTBP1. We used the NMR solution structure for PTBP2 to determine the location of these residues (Fig 8A). We used the NMR solution structure of PTBP1 bound to the CUCUCU RNA hexamer to determine the interactions (if any) of these lysine side chains with the RNA substrate. Lys436 is located on a flexible loop region and has the potential to make H-bond interactions with the C3 nitrogenous base. Importantly, the positively charged **ε-**amino group is located for optimal phi-cation interactions with the C3 base (Fig 8B). Thus, acetylation at this side chain which will remove the positive charge and can disrupt this interaction. Lys454 is pointing away from the RNA interaction surface of the ß sheet and may play a role in protein-protein interactions. PTBP1 Lys 400 (counterpart of PTBP2 Lys375) is located in the loop region between ß strands 2 and 3 pointing towards the bound RNA and in close proximity highlighting the potential for interacting with the RNA via H-bonds. However, the distances between H-bond forming atoms are larger than 3 Å in the solution structure (Fig 8B).

PTBP1 but not PTBP2 is acetylated at two positions in RRM4 at Lys497 and Lys508. Neither of these lysine side chains make direct interaction with the bound RNA via the **ε-**amino groups (Fig 8B). Our results highlight that PTBP RRMs 3 and 4 are less modified (and therefore less modulated) by acetylation compared to RRMs 1 and 2 in neuronal nuclear extract.

Overall, our data reveal PTBP1 is acetylated at many more residues compared to PTBP2 under neuronal splicing conditions. However, in contrast to HeLa nuclear extract [23], we note a higher number of acetyl modifications localized to the RRMs 1 and 2 regions in neuronal extract for PTBP1. PTBP1 has less acetyl modifications in RRMs 3 and 4 under neuronal nuclear extract.

### The PTBP paralogs are modified by the addition of methyl groups in neuronal nuclear extract

A novel finding from our chemical modification study is the paralogs are modified by methylation under WERI nuclear extract. Moreover, PTBP2 but not PTBP1 is also dimethylated under these conditions. Protein arginine methyltransferases (PRMT) and Protein lysine methyltransferases (PKMT) catalyze the methylation of these amino acids (Fig 6B left and 6C) [55,56,59]. Methylation increases the hydrophobicity of the lysine side chain that remains positively charged. PTBP1 is methylated at Lys45 which is part of the nuclear localization sequence in the N terminal region (Table 1). PTBP2 is dimethylated at Arg14 which is also part of both the nuclear export and import sequences (Table 2) [44,48]. The presence of acetyl and methyl groups in the NLS sequence in addition to the previously reported phosphate modification (at Ser16) in this region [60] suggest a complex regulation of PTBP1 localization in response to cellular signals and metabolic state. This notion will be addressed in future work.

The PTB proteins are methylated in the RRM1 region in both paralogs (Tables 1 and 2). Overall, PTBP1 has many more sites of methylation compared to PTBP2. We note significant overlap between residues that are modified by acetyl and methyl groups in PTBP1 in this region. For PTBP1, Lys65, Lys84, Lys92, Lys94, Lys134 and Lys137 carry both modifications. The paralogs have over-lapping methyl modifications at residues Lys94, Arg122 and Lys134. Lys65 is located in the turn between ß1 and α1 (Fig 7A). It is positioned in close proximity to the ribose sugar C5-hydroxyl group, yet the amino group is pointing away and does not make H-bond interactions. Lys 84, Lys92, Arg122 and Lys 134 are pointed away from the RNA substrate and can likely participate in protein-protein interactions including methyl lysine reader proteins such as TUDOR (S4 Table) (Fig 7A). The **ε-**amino group of Lys94 contacts the phosphodiester linkage between C5 and U6 via a hydrogen bond interaction. Depending on the position of the methyl group this H-bond interaction can be disrupted. Lys137 makes an H-bond interaction with the nitrogenous base of U4 that can be disrupted by methylation. Arg 122 is located in a flexible loop located between **α** helix 2 and ß 4 and is not in H-bond distance to the RNA substrate but can likely participate in mediating protein-protein interactions. Notably, all PTBP1 acetylated lysine side chains are also modified via methylation. Arg122 is the only arginine residue that is methylated. In contrast, for PTBP2, only one (Lys134) out of the four residues have overlapping acetyl-methyl modifications. Thus, our data support a role for cross talk between acetylation and methylation regulators in modulating RRM1 and in turn PTBP1 function in response to cellular needs.

In RRM2, we observe overlapping methyl modifications between the paralogs at PTBP1 residues Arg 254 (PTBP2 Arg 251), Arg 277 (PTBP2 Arg274) and PTBP1 Lys238 (PTBP2 Lys235). PTBP1 is distinctly monomethylated at Lys206, Lys218 (also acetylated) and Arg185. PTBP2 is distinctly methylated at Lys268. Notably, PTBP2 is dimethylated at Arg182 (PTBP1 counterpart Arg185 is monomethylated), Arg270 and Arg274 (also monomethylated). We utilized the solution structure of PTBP1 RRM2 to determine the position and interactions of these residues in the RNA binding domain with the bound CUCUCU RNA hexamer. The Arg185 guanidino group (PTBP2 counterpart Arg182) makes an H-bond interaction with the ribose sugar of U2. Methylation at this position including dimethylation (in PTBP2) can disrupt this H-bond and in turn, RNA binding affinity. Arg185 is well-positioned to participate in phi-cation interactions with the U2 base (Fig 7B). Methylation of the guanidino group and inductive effects from the methyl group(s) will stabilize the positive charge on the guanidino group and may decrease this stabilizing interaction.

Lys206 is located on the α helical side of the domain and can participate in protein-protein interactions. Lys218 makes three H-bond interactions with the phosphodiester linkage between C3 and U4. Addition of a methyl group at this position can impact one or more H-bonds (dependent on steric bulk) and in turn, RNA binding affinity. Lys238 (PTBP2 Lys235) is located on the α helical side of the domain and can play a role in protein-protein interactions. We note this residue lies close to the Raver 1 interacting motif (Fig 1C). PTBP1 but not PTBP2 interacts with the Raver1 protein in vivo albeit the near-identical interacting sequence motif. We note PTBP1 Lys 238 is both acetylated and methylated while PTBP2 counterpart (Lys 235) is only methylated raising the notion that acetylation at this position may play a role in modulating PTBP1 interaction with Raver1. Arg254 (PTBP2 counterpart Arg251) is pointing away from the RNA interaction surface but is in close proximity to the Raver 1 interacting motif. Thus, methylation at this side chain may play a role in modulating protein-protein interactions. Arg277 is in a flexible loop region pointing away from the RNA interaction surface. We surmise methylation at this side chain (based on available structural information) does not play a direct role in RNA binding affinity. PTBP2 Lys268 (PTBP1 counterpart Lys271) makes an H-bond interaction with the ribose sugar moiety of U6. PTBP2 Arg 270 (PTBP1 counterpart Arg 273) and Arg274 (PTBP1 counterpart Arg274) are dimethylated. These two residues are pointing away from the RNA interaction surface and ideally located to participate in protein-protein interactions (Fig 7B). Overall, our data support a role for RRM2 methylation in regulating RRM2-mediated PTBP RNA binding affinity and protein-protein interactions.

Our data indicate that PTBP2 RRM3 region residues Lys375, and Lys429 are monomethylated (and have over-lapping acetyl modifications) and Arg 419 is dimethylated (Table 2). We used the PTBP2 RRM3-RRM4 solution structure to analyze the position and interactions of these residues (Fig 8A). Lys375 is located in the loop connecting ß strands 2 and 3. This residue has the potential to participate in RNA binding. PTBP1 counterpart Lys400 is near the C5 nitrogenous base and the phosphodiester bond between C3 and U4 and may participate in H-bond interactions (although bond distance in over 3.5 Å). Thus, methylation at PTBP2 Lys375 may play a role in RNA binding affinity. Lys429 (PTBP1 Lys454) is situated away from the RNA interacting ß sheet surface and does not interact with the bound RNA. Arg 419 (PTBP1 Arg444) is located in a flexible loop region connecting ß strands 4 and 5. This residue is pointing towards the bound CUCUCU hexamer and is in close proximity to the ribose sugar of U6. Given the flexibility of this loop region, Arg 419 has the potential to participate in stabilizing phi-cation interactions with the U6 base. Thus, dimethylation at this position can play a role in PTBP2 RNA binding affinity.

PTBP1 is methylated at distinct residues Arg374, Arg392, Lys424 and Lys436. Arg374 is located on the flexible loop region between ß strand 1 and α helix 1. This residue is pointing away from the RNA interacting surface and it located in an ideal position to participate in protein-protein interactions. Arg392 is located on ß strand 2 but does not make any interactions with the bound RNA. The **ε-**amino group of Lys424 makes an H-bond interaction with Glu502 in RRM4. This interaction plays an important role in maintaining the unique structural arrangement of RRM3 and 4 [16]. Thus, methylation at Lys424 can perturb this interaction and play a role in PTBP1 function. Lys436 is located in the flexible loop region between ß strands 4 and 5. Depending on the positioning of this loop the **ε-**amino group can interact with the C3 nitrogenous base and the phosphodiester linkage between U2 and C3 via H-bonding and can participate in phi-cation interactions. Thus, methylation at this position can influence RNA binding affinity. Collectively, our data suggest that methylation in RRM3 region can alter PTBP1 properties including RNA binding and protein-protein interactions. Moreover, we note that PTBP1 RRM3 region is modified to a greater extent via methylation than acetylation in neuronal nuclear extract. This result contrast with previous findings where PTBP1 RRM3 region was acetylated under HeLa nuclear extract. Thus, our data reveal PTBP1 is modified differently in the two distinct types of extract in the RRM3 region.

The paralogs share over-lapping methyl modifications in the RRM4 region. PTBP2 is modified at Arg472 (PTBP1 counterpart Lys497) and Lys 483 (PTBP1 counterpart Ly508). For PTBP1, Lys497 is located on α helix 2 and can participate in protein-protein interactions. Lys508 is located on the ß sheet surface but does not make interactions with the bound RNA (Fig 8B). PTBP2 methylated residues are also positioned away from the RNA interacting surface (Fig 8A). Thus, PTBP methylation in the RRM4 region likely does not play a role in RNA binding but may play a role in protein-protein interactions.

## Discussion

Our studies highlight the paralogs have distinct interacting partner proteins under neuronal splicing conditions. Notably, our protein-protein interaction data reveal that PTBP2 but not PTBP1 interacts with splicing factors in the catalytic spliceosome. PTBP1 co-purified with proteins associated with ribosome structure and translation signifying distinct preferences exhibited by the paralogs under neuronal nuclear extract. During neuronal differentiation and maturation, the levels of PTBP1 go down while that of PTBP2 goes up to mediate distinct splicing outcomes in neuronal transcripts that are critical for differentiation and maturation [9,10,61]. How the two proteins can exert distinct splicing outcomes is not understood given their high similarity in primary and tertiary structure. Our data support a role for differences in protein-protein interactions in mediating the paralogs’ ability to distinctly regulate neuronal transcripts. Furthermore, our chemical modification data reveal that albeit high primary structure identity, the paralogs are differentially modified by the addition of acetyl, methyl and phosphate groups under neuronal nuclear extract. Our findings signify and reiterate the PTBP2 N-terminal region is phosphorylated to a significant extent under both neuronal and non-neuronal conditions suggesting a yet-to-be discovered novel role for this region and phosphorylation in PTBP2 function. It is noteworthy that acetyl and methyl modifications occur on the same lysine side chains indicating the presence of cross talk between cell signaling pathways modulating PTBP function. Whether these modifications play a role in PTBP-regulated alternative splicing activity is an important question that will be addressed in future studies.

Additionally, arginine methylation alters nucleocytoplasmic shuttling of proteins [62,63]. RNA binding proteins account for 60% of the asymmetric dimethylated arginines found in the nucleus [64,65]. The mechanism by which arginine methylation regulates shuttling is not understood. Many RBPs have been highlighted as methylated. Components of the spliceosome and hnRNP are methylated at arginine residues indicating a role for this modification in regulating splicing [66–68]. Arginine methylation may positively regulate RNA-protein interactions as the arginine becomes more hydrophobic with methyl groups and this can facilitate stacking interactions with the nitrogenous bases of RNA. Additionally, steric bulk and removal of H-bonds may play a role in protein-RNA interactions as well.

Collectively, our findings highlight important differences in protein-protein interactions that likely dictate the PTB paralogs distinct splicing activities. Our results also suggest post-translational modifications as candidates that mediate the paralogs distinct splicing activities via modulating protein-protein and protein-RNA interactions. Future studies will address this notion to further our understanding of how related members in a gene family can dictate tissue-specific gene expression patterns.

## Supporting information

S1 Fig Log fractional difference of observed vs expected PANTHER GO-Slim molecular function categories assigned to proteins found unique to the PTBP2 pulldown. The log fractional difference is calculated for each category as (# genes for the category-# genes expected)/ # genes expected) as provided on Gene Ontology. Highest functions on this graph include histone deacetylase binding, transcription regulation, and RNA binding.
(TIF)

S2 Fig Log fractional difference of observed vs expected PANTHER GO-Slim molecular function categories assigned to proteins found unique to the PTBP1 pulldown. The log fractional difference is calculated for each category as (# genes for the category-# genes expected)/ # genes expected) as provided on Gene Ontology. Highest functions on this graph include structural constituent of ribosome and RNA binding, implying function in both mRNA processing and translation.
(TIF)

S3 Fig Experimental workflow diagram. A flow chart that outlines the steps carried out to determine protein-protein interactions and post-translational modifications in the PTB proteins.
(PDF)

S1 Table Proteins that co-purified and were unique to PTBP1 incubated in Buffer DG. Recombinant His-tagged PTBP1 purified via nickel affinity chromatography was incubated in WERI retinoblastoma nuclear extract. Proteins listed in this table may have co-purified during recombinant expression and purification of His-tagged PTBP1.
(PDF)

S2 Table Proteins that co-purified and were unique to PTBP2 incubated in Buffer DG. Recombinant His-tagged PTBP2 purified vai nickel affinity chromatography was incubated in WERI retinoblastoma nuclear extract. Proteins listed in this table may have co-purified during recombinant expression and purification of His-tagged PTBP2.
(PDF)

S3 Table Proteins in WERI nuclear extract that interact unspecifically with the Ni^2+^ magnetic beads. Proteins in the WERI nuclear extract that interacted unspecifically with the beads are listed in this table.
(PDF)

S4 Table Unique proteins that associate with both PTBP1 and PTBP2 under neuroanl WERI nuclear extract conditions. Unique proteins that either directly or indirectly (via bound RNA) interact and co-elute with both PTBP1 and PTBP2 are listed in this table. Proteins identified as unspecifically bound to the Ni2 + beads and carried over during recombinant expression and purification were removed from this list.
(PDF)

S5 Table Unique proteins that associate with PTBP2 under neuronal WERI nuclear extract conditions. Unique proteins that interact and co-elute with PTBP2 are listed in this table. Proteins identified as unspecifically bound to the Ni2 + beads and carried over during recombinant expression and purification have been removed from this list.
(PDF)

S6 Table Unique proteins that associate with PTBP1 under neuronal WERI nuclear extract conditions. Unique proteins that interact and co-elute with PTBP1 are listed in this table. Proteins identified as unspecifically bound to the Ni2 + beads and carried over during recombinant expression and purification have been removed from this list.
(PDF)
