# Supplementary figures and images for: Polypyrimidine tract binding proteins PTBP1 and PTBP2 associate with distinct proteins and have distinct post-translational modifications in neuronal nuclear extract
Source: PLoS One. 2025 Jun 4;20(6):e0325143. doi: 10.1371/journal.pone.0325143 (PMC12136456; doi:10.1371/journal.pone.0325143)

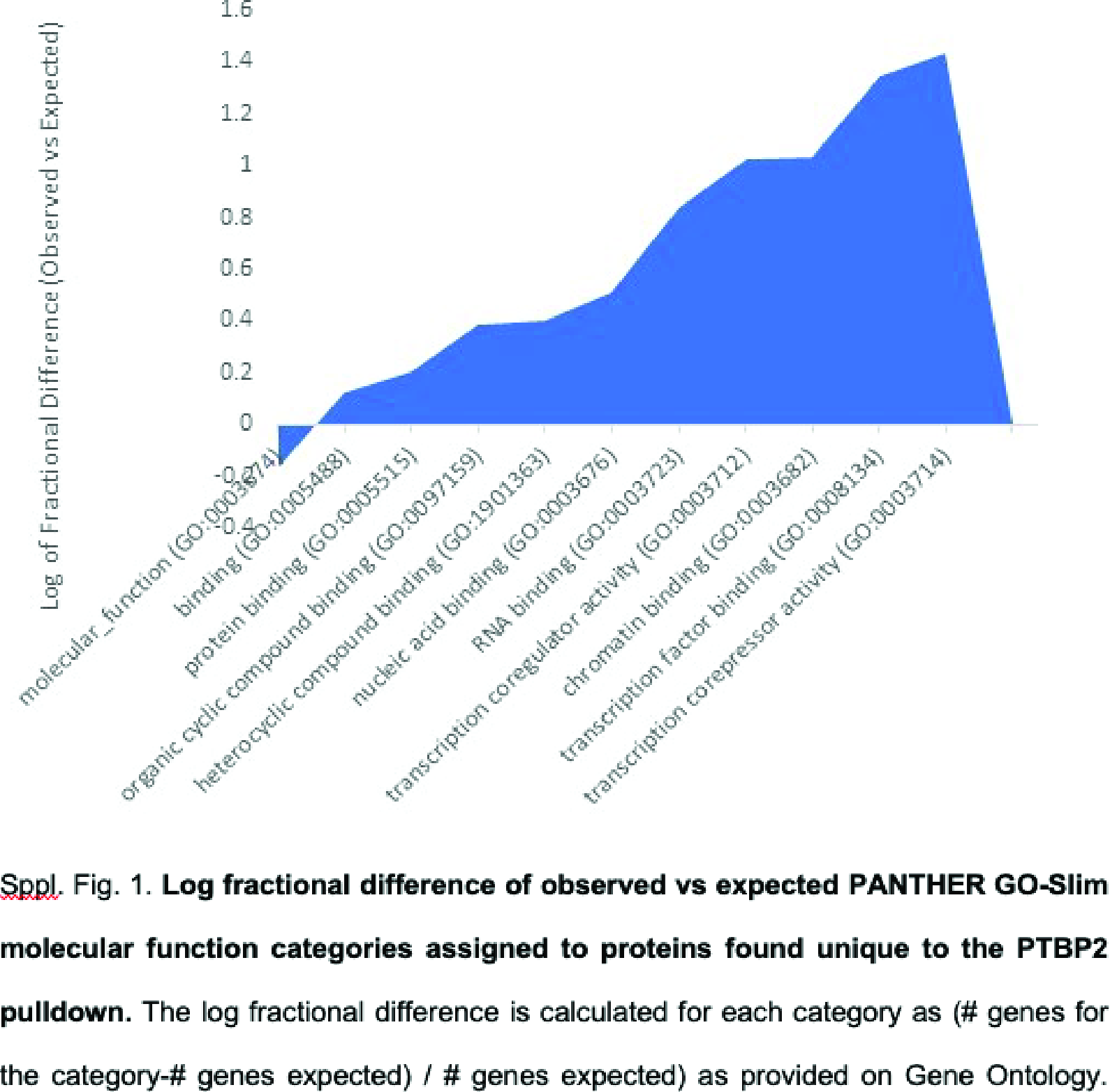

Supplement: S1 Fig — The log fractional difference is calculated for each category as (# genes for the category-# genes expected)/ # genes expected) as provided on Gene Ontology. Highest functions on this graph include histone deacetylase binding, transcription regulation, and RNA binding. (TIF) [file pone.0325143.s001.tif]

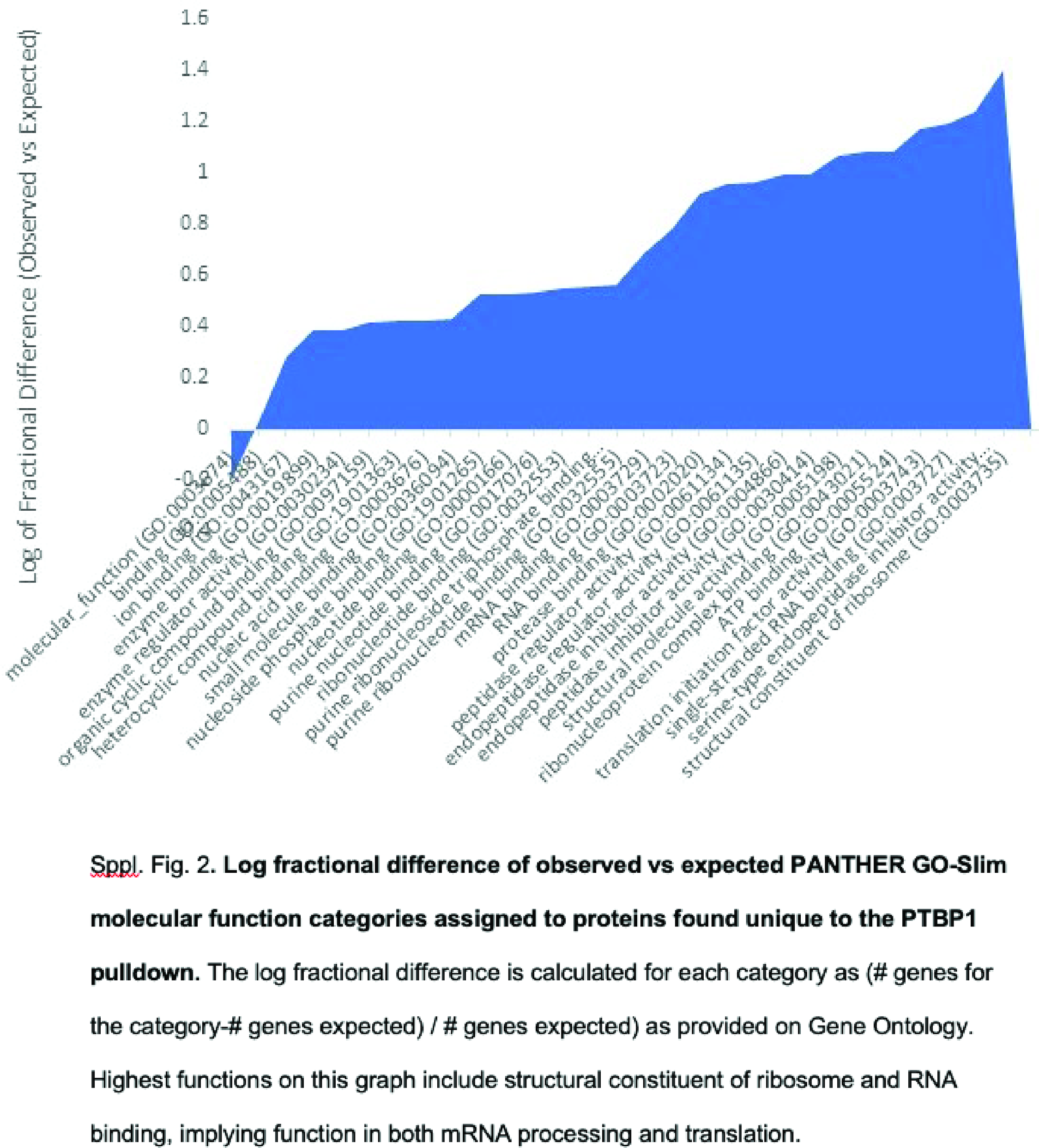

Supplement: S2 Fig — The log fractional difference is calculated for each category as (# genes for the category-# genes expected)/ # genes expected) as provided on Gene Ontology. Highest functions on this graph include structural constituent of ribosome and RNA binding, implying function in both mRNA processing and translation. (TIF) [file pone.0325143.s002.tif]
