# Supplementary material for: Polypyrimidine tract binding proteins PTBP1 and PTBP2 associate with distinct proteins and have distinct post-translational modifications in neuronal nuclear extract
Source: PLoS One. 2025 Jun 4;20(6):e0325143. doi: 10.1371/journal.pone.0325143 (PMC12136456; doi:10.1371/journal.pone.0325143)
Supplement: S3 Fig — A flow chart that outlines the steps carried out to determine protein-protein interactions and post-translational modifications in the PTB proteins. (PDF) [file pone.0325143.s003.pdf]

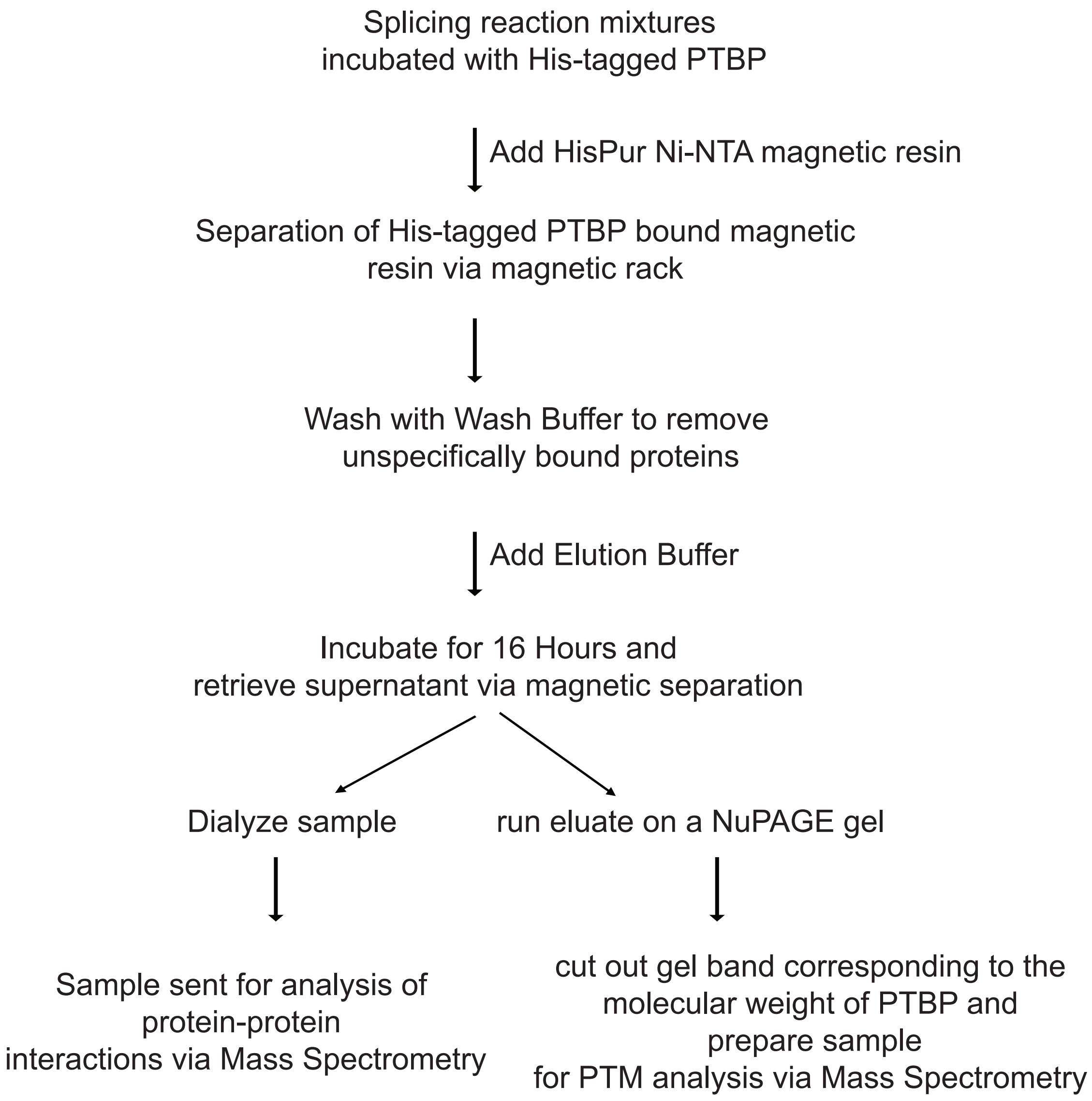
Splicing reaction mixtures
incubated with His-tagged PTBP
Add HisPur Ni-NTA magnetic resin
Separation of His-tagged PTBP bound magnetic
resin via magnetic rack
Wash with Wash Buffer to remove
unspecifically bound proteins
Add Elution Buffer
Incubate for 16 Hours and
retrieve supernatant via magnetic separation
Dialyze sample
run eluate on a NuPAGE gel
Sample sent for analysis of
protein-protein
interactions via Mass Spectrometry
cut out gel band corresponding to the
molecular weight of PTBP and
prepare sample
for PTM analysis via Mass Spectrometry
