# Supplementary material for: Polypyrimidine tract binding proteins PTBP1 and PTBP2 associate with distinct proteins and have distinct post-translational modifications in neuronal nuclear extract
Source: PLoS One. 2025 Jun 4;20(6):e0325143. doi: 10.1371/journal.pone.0325143 (PMC12136456; doi:10.1371/journal.pone.0325143)
Supplement: S1 Table — Recombinant His-tagged PTBP1 purified via nickel affinity chromatography was incubated in WERI retinoblastoma nuclear extract. Proteins listed in this table may have co-purified during recombinant expression and purification of His-tagged PTBP1. (PDF) [file pone.0325143.s004.pdf]

Sppl. Table. 1. Proteins that co-purified and were unique to PTBP1 incubated in Buffer DG.

Accession(PTBP1 DG)	Gene Name	Description
F5H0C8|F5H0C8_HUMAN	ENO2	2-phospho-D-glycerate hydro-lyase OS=Homo sapiens OX=9606 GN=ENO2 PE=1 SV=1
P15104|GLNA_HUMAN	GLUL	Glutamine synthetase OS=Homo sapiens OX=9606 GN=GLUL PE=1 SV=4
P08708|RS17_HUMAN	RPS17	40S ribosomal protein S17 OS=Homo sapiens OX=9606 GN=RPS17 PE=1 SV=2
A0A804F6T5|A0A804F6T5_HUMAN	PKM	Pyruvate kinase PKM OS=Homo sapiens OX=9606 GN=PKM PE=4 SV=1
A0A6Q8PFK8|A0A6Q8PFK8_HUMAN	HSPB1	Heat shock protein beta-1 OS=Homo sapiens OX=9606 GN=HSPB1 PE=1 SV=1
Q5CZC0|FSIP2_HUMAN	FSIP2	Fibrous sheath-interacting protein 2 OS=Homo sapiens OX=9606 GN=FSIP2 PE=2 SV=4
A8MX12|A8MX12_HUMAN	MYOM1	Myomesin-1 OS=Homo sapiens OX=9606 GN=MYOM1 PE=1 SV=3
Q14CN4|K2C72_HUMAN	KRT72	Keratin type II cytoskeletal 72 OS=Homo sapiens OX=9606 GN=KRT72 PE=1 SV=2
Q15517|CDSN_HUMAN	CDSN	Corneodesmosin OS=Homo sapiens OX=9606 GN=CDSN PE=1 SV=3
A0A7P0TB36|A0A7P0TB36_HUMAN	HSPA5	78 kDa glucose-regulated protein OS=Homo sapiens OX=9606 GN=HSPA5 PE=1 SV=1
A0A6Q8PFK6|A0A6Q8PFK6_HUMAN	EEF1A2	Elongation factor 1-alpha 2 OS=Homo sapiens OX=9606 GN=EEF1A2 PE=4 SV=1
M0R1V7|M0R1V7_HUMAN	UBA52	Ubiquitin-60S ribosomal protein L40 (Fragment) OS=Homo sapiens OX=9606 GN=UBA52 PE=1 S[illegible]
P0CG48|UBC_HUMAN	UBC	Polyubiquitin-C OS=Homo sapiens OX=9606 GN=UBC PE=1 SV=3
J3QSA3|J3QSA3_HUMAN	UBB	Polyubiquitin-B (Fragment) OS=Homo sapiens OX=9606 GN=UBB PE=1 SV=1
P18859|ATP5J_HUMAN	ATP5PF	ATP synthase-coupling factor 6 mitochondrial OS=Homo sapiens OX=9606 GN=ATP5PF PE=1 SV[illegible]
Q6IS14|IF5AL_HUMAN	EIF5AL1	Eukaryotic translation initiation factor 5A-1-like OS=Homo sapiens OX=9606 GN=EIF5AL1 PE=2 [illegible]
I3L504|I3L504_HUMAN	EIF5A	Eukaryotic translation initiation factor 5A-1 OS=Homo sapiens OX=9606 GN=EIF5A PE=1 SV=1
A0A024R6I7|A0A024R6I7_HUMAN	SERPINA1	Alpha-1-antitrypsin OS=Homo sapiens OX=9606 GN=SERPINA1 PE=1 SV=1
Q6S8J3|POTEE_HUMAN	POTEE	POTE ankyrin domain family member E OS=Homo sapiens OX=9606 GN=POTEE PE=2 SV=3
H0YLE2|H0YLE2_HUMAN	ANXA2	Annexin A2 (Fragment) OS=Homo sapiens OX=9606 GN=ANXA2 PE=1 SV=1
A6NMY6|AXA2L_HUMAN	ANXA2P2	Putative annexin A2-like protein OS=Homo sapiens OX=9606 GN=ANXA2P2 PE=5 SV=2
Q96NL0|RUN3B_HUMAN	RUNDC3B	RUN domain-containing protein 3B OS=Homo sapiens OX=9606 GN=RUNDC3B PE=2 SV=1

V=1

'=1
;V=2
