# Supplementary material for: Polypyrimidine tract binding proteins PTBP1 and PTBP2 associate with distinct proteins and have distinct post-translational modifications in neuronal nuclear extract
Source: PLoS One. 2025 Jun 4;20(6):e0325143. doi: 10.1371/journal.pone.0325143 (PMC12136456; doi:10.1371/journal.pone.0325143)
Supplement: S2 Table — Recombinant His-tagged PTBP2 purified vai nickel affinity chromatography was incubated in WERI retinoblastoma nuclear extract. Proteins listed in this table may have co-purified during recombinant expression and purification of His-tagged PTBP2. (PDF) [file pone.0325143.s005.pdf]

Sppl. Table. 2. Proteins that co-purified and were unique to PTBP2 incubated in Buffer DG.

Accession(PTBP2 DG)	Gene Nam	Description
K7ERE3|K7ERE3_HUMAN	KRT13	Keratin type I cytoskeletal 13 OS=Homo sapiens OX=9606 GN=KRT13 PE=1 SV=1
E9PLF4|E9PLF4_HUMAN	HSPA8	Heat shock cognate 71 kDa protein (Fragment) OS=Homo sapiens OX=9606 GN=HSPA8 PE=1 SV=1
P54652|HSP72_HUMAN	HSPA2	Heat shock-related 70 kDa protein 2 OS=Homo sapiens OX=9606 GN=HSPA2 PE=1 SV=1
Q53FA3|Q53FA3_HUMAN	HSPA1L	HSPA1L (Fragment) OS=Homo sapiens OX=9606 GN=HSPA1L PE=1 SV=1
G3V1R1|G3V1R1_HUMAN	PRB1	Basic salivary proline-rich protein 1 OS=Homo sapiens OX=9606 GN=PRB1 PE=1 SV=1
P02812|PRB2_HUMAN	PRB2	Basic salivary proline-rich protein 2 OS=Homo sapiens OX=9606 GN=PRB2 PE=1 SV=3
