# Supplementary material for: Polypyrimidine tract binding proteins PTBP1 and PTBP2 associate with distinct proteins and have distinct post-translational modifications in neuronal nuclear extract
Source: PLoS One. 2025 Jun 4;20(6):e0325143. doi: 10.1371/journal.pone.0325143 (PMC12136456; doi:10.1371/journal.pone.0325143)
Supplement: S3 Table — Proteins in the WERI nuclear extract that interacted unspecifically with the beads are listed in this table. (PDF) [file pone.0325143.s006.pdf]

Sppl. Table. 3. Proteins in WERI nuclear extract that bound unspecifically to the Ni^{2+} magnetic beads

Accession (WERI only control)	Gene Name	Description
tr|E9PES6|E9PES6_HUMAN	HMGB3	High mobility group protein B3 (Fragment) OS=Homo sapiens OX=9606 GN=HMGB3 PE=1 SV=1
Q14194|DPYL1_HUMAN	CRMP1	Dihydropyrimidinase-related protein 1 OS=Homo sapiens OX=9606 GN=CRMP1 PE=1 SV=1
tr|F5H018|F5H018_HUMAN	RAN	GTP-binding nuclear protein Ran (Fragment) OS=Homo sapiens OX=9606 GN=RAN PE=1 SV=8
tr|E9PK25|E9PK25_HUMAN	CFL1	Cofilin non-muscle isoform OS=Homo sapiens OX=9606 GN=CFL1 PE=1 SV=1
A0A087WTP3_HUMAN	KHSRP	Far upstream element-binding protein 2 OS=Homo sapiens OX=9606 GN=KHSRP PE=1 SV=1
O14497|ARI1A_HUMAN	ARID1A	AT-rich interactive domain-containing protein 1A OS=Homo sapiens OX=9606 GN=ARID1A PE=1 SV=3
O60828|PQBP1_HUMAN	PQBP1	Polyglutamine-binding protein 1 OS=Homo sapiens OX=9606 GN=PQBP1 PE=1 SV=1
Q9BXP5|SRRT_HUMAN	SRRT	Serrate RNA effector molecule homolog OS=Homo sapiens OX=9606 GN=SRRT PE=1 SV=1
Q15691|MARE1_HUMAN	MAPRE1	Microtubule-associated protein RP/EB family member 1 OS=Homo sapiens OX=9606 GN=MAPRE1 PE=1 SV=3
A0A7I2V2R3_HUMAN	HNRNPA3	Heterogeneous nuclear ribonucleoprotein A3 OS=Homo sapiens OX=9606 GN=HNRNPA3 PE=1 SV=1
A0A7I2V4F0_HUMAN	DDX1	ATP-dependent RNA helicase DDX1 OS=Homo sapiens OX=9606 GN=DDX1 PE=1 SV=1
Q9Y3I0|RTCB_HUMAN	RTCB	RNA-splicing ligase RtcB homolog OS=Homo sapiens OX=9606 GN=RTCB PE=1 SV=1
A0A2R8YD58_HUMAN	CSNK2A1	Casein kinase II subunit alpha OS=Homo sapiens OX=9606 GN=CSNK2A1 PE=1 SV=1
Q08945|SSRP1_HUMAN	SSRP1	FACT complex subunit SSRP1 OS=Homo sapiens OX=9606 GN=SSRP1 PE=1 SV=1
P78347|GTF2I_HUMAN	GTF2I	General transcription factor II-I OS=Homo sapiens OX=9606 GN=GTF2I PE=1 SV=2
P35637|FUS_HUMAN	FUS	RNA-binding protein FUS OS=Homo sapiens OX=9606 GN=FUS PE=1 SV=1
Q9BTA9|WAC_HUMAN	WAC	WW domain-containing adapter protein with coiled-coil OS=Homo sapiens OX=9606 GN=WAC PE=1 SV=3
P28482|MK01_HUMAN	MAPK1	Mitogen-activated protein kinase 1 OS=Homo sapiens OX=9606 GN=MAPK1 PE=1 SV=3
P84103|SRSF3_HUMAN	SRSF3	Serine/arginine-rich splicing factor 3 OS=Homo sapiens OX=9606 GN=SRSF3 PE=1 SV=1
Q96GM5|SMRD1_HUMAN	SMARCD1	SWI/SNF-related matrix-associated actin-dependent regulator of chromatin subfamily D member 1 OS=Homo sapiens OX
P09234|RU1C_HUMAN	SNRPC	U1 small nuclear ribonucleoprotein C OS=Homo sapiens OX=9606 GN=SNRPC PE=1 SV=1
P12277|KCRB_HUMAN	CKB	Creatine kinase B-type OS=Homo sapiens OX=9606 GN=CKB PE=1 SV=1
P27695|APEX1_HUMAN	APEX1	DNA-(apurinic or apyrimidinic site) endonuclease OS=Homo sapiens OX=9606 GN=APEX1 PE=1 SV=2
Q96P16|RPR1A_HUMAN	RPRD1A	Regulation of nuclear pre-mRNA domain-containing protein 1A OS=Homo sapiens OX=9606 GN=RPRD1A PE=1 SV=1
P51858|HDGF_HUMAN	HDGF	Hepatoma-derived growth factor OS=Homo sapiens OX=9606 GN=HDGF PE=1 SV=1
tr|K7EJB8|K7EJB8_HUMAN	PPP1R14A	Protein phosphatase 1 regulatory subunit 14A OS=Homo sapiens OX=9606 GN=PPP1R14A PE=1 SV=1
Q96CN7|ISOC1_HUMAN	ISOC1	Isochorismatase domain-containing protein 1 OS=Homo sapiens OX=9606 GN=ISOC1 PE=1 SV=3
O43670|ZN207_HUMAN	ZNF207	BUB3-interacting and GLEBS motif-containing protein ZNF207 OS=Homo sapiens OX=9606 GN=ZNF207 PE=1 SV=1
tr|G3V555|G3V555_HUMAN	HNRNPC	Heterogeneous nuclear ribonucleoproteins C1/C2 (Fragment) OS=Homo sapiens OX=9606 GN=HNRNPC PE=1 SV=1
A0A0A0MTS7_HUMAN	TTN	Titin OS=Homo sapiens OX=9606 GN=TTN PE=1 SV=1
tr|Q7Z3H1|Q7Z3H1_HUMAN	AIPL1	Aryl-hydrocarbon-interacting protein-like 1 OS=Homo sapiens OX=9606 GN=AIPL1 PE=1 SV=1
Q9Y5B9|SP16H_HUMAN	SUPT16H	FACT complex subunit SPT16 OS=Homo sapiens OX=9606 GN=SUPT16H PE=1 SV=1

P22392|NDKB_HUMAN	NME2	Nucleoside diphosphate kinase B OS=Homo sapiens OX=9606 GN=NME2 PE=1 SV=1
tr|Q32Q12|Q32Q12_HUMAN	NME1-NME2	Nucleoside diphosphate kinase OS=Homo sapiens OX=9606 GN=NME1-NME2 PE=1 SV=1
O60361|NDK8_HUMAN	NME2P1	Putative nucleoside diphosphate kinase OS=Homo sapiens OX=9606 GN=NME2P1 PE=5 SV=1
tr|E7ERL0|E7ERL0_HUMAN	NME1	Nucleoside diphosphate kinase A OS=Homo sapiens OX=9606 GN=NME1 PE=1 SV=1
Q04726|TLE3_HUMAN	TLE3	Transducin-like enhancer protein 3 OS=Homo sapiens OX=9606 GN=TLE3 PE=1 SV=2
Q9Y314|NOSIP_HUMAN	NOSIP	Nitric oxide synthase-interacting protein OS=Homo sapiens OX=9606 GN=NOSIP PE=1 SV=1
tr|H0Y6E7|H0Y6E7_HUMAN	RBMX	RNA-binding motif protein X chromosome (Fragment) OS=Homo sapiens OX=9606 GN=RBMX PE=1 SV=2
Q96E39|RMXL1_HUMAN	RBMXL1	RNA binding motif protein X-linked-like-1 OS=Homo sapiens OX=9606 GN=RBMXL1 PE=1 SV=1
O75526|RMXL2_HUMAN	RBMXL2	RNA-binding motif protein X-linked-like-2 OS=Homo sapiens OX=9606 GN=RBMXL2 PE=1 SV=3
Q8N7X1|RMXL3_HUMAN	RBMXL3	RNA-binding motif protein X-linked-like-3 OS=Homo sapiens OX=9606 GN=RBMXL3 PE=2 SV=2
Q13394|MB211_HUMAN	MAB21L1	Putative nucleotidyltransferase MAB21L1 OS=Homo sapiens OX=9606 GN=MAB21L1 PE=1 SV=1
P48443|RXRG_HUMAN	RXRG	Retinoic acid receptor RXR-gamma OS=Homo sapiens OX=9606 GN=RXRG PE=1 SV=1
P09661|RU2A_HUMAN	SNRPA1	U2 small nuclear ribonucleoprotein A' OS=Homo sapiens OX=9606 GN=SNRPA1 PE=1 SV=2
Q8IZ73|RUSD2_HUMAN	RPUSD2	RNA pseudouridylate synthase domain-containing protein 2 OS=Homo sapiens OX=9606 GN=RPUSD2 PE=1 SV=2
Q9Y5V0|ZN706_HUMAN	ZNF706	Zinc finger protein 706 OS=Homo sapiens OX=9606 GN=ZNF706 PE=1 SV=1
Q6UXN9|WDR82_HUMAN	WDR82	WD repeat-containing protein 82 OS=Homo sapiens OX=9606 GN=WDR82 PE=1 SV=1
A0A7P0Z497_HUMAN	PPIB	Peptidyl-prolyl cis-trans isomerase OS=Homo sapiens OX=9606 GN=PPIB PE=1 SV=1
Q6NZY4|ZCHC8_HUMAN	ZCCHC8	Zinc finger CCHC domain-containing protein 8 OS=Homo sapiens OX=9606 GN=ZCCHC8 PE=1 SV=2
A0A3B3ITT1_HUMAN	RAB12	Ras-related protein Rab-12 OS=Homo sapiens OX=9606 GN=RAB12 PE=1 SV=1
Q52LJ0|FA98B_HUMAN	FAM98B	Protein FAM98B OS=Homo sapiens OX=9606 GN=FAM98B PE=1 SV=2
O95347|SMC2_HUMAN	SMC2	Structural maintenance of chromosomes protein 2 OS=Homo sapiens OX=9606 GN=SMC2 PE=1 SV=2
tr|F8WE41|F8WE41_HUMAN	NCBP2	Nuclear cap-binding protein subunit 2 OS=Homo sapiens OX=9606 GN=NCBP2 PE=1 SV=1
Q92785|REQU_HUMAN	DPF2	Zinc finger protein ubi-d4 OS=Homo sapiens OX=9606 GN=DPF2 PE=1 SV=2
A0A0A0MRE5_HUMAN	ASAP1	Arf-GAP with SH3 domain ANK repeat and PH domain-containing protein 1 OS=Homo sapiens OX=9606 GN=ASAP1 PE=1
P31146|COR1A_HUMAN	CORO1A	Coronin-1A OS=Homo sapiens OX=9606 GN=CORO1A PE=1 SV=4
tr|H3BNI9|H3BNI9_HUMAN	CSNK2A2	Casein kinase II subunit alpha' OS=Homo sapiens OX=9606 GN=CSNK2A2 PE=1 SV=2
O15020|SPTN2_HUMAN	SPTBN2	Spectrin beta chain non-erythrocytic 2 OS=Homo sapiens OX=9606 GN=SPTBN2 PE=1 SV=3
A0A2R8Y706_HUMAN	DYNC1H1	Cytoplasmic dynein 1 heavy chain 1 OS=Homo sapiens OX=9606 GN=DYNC1H1 PE=1 SV=2
Q8N1G2|CMTR1_HUMAN	CMTR1	Cap-specific mRNA (nucleoside-2'-O-)-methyltransferase 1 OS=Homo sapiens OX=9606 GN=CMTR1 PE=1 SV=1
P10588|NR2F6_HUMAN	NR2F6	Nuclear receptor subfamily 2 group F member 6 OS=Homo sapiens OX=9606 GN=NR2F6 PE=1 SV=2
Q4LE39|ARI4B_HUMAN	ARID4B	AT-rich interactive domain-containing protein 4B OS=Homo sapiens OX=9606 GN=ARID4B PE=1 SV=2
P53396|ACLY_HUMAN	ACLY	ATP-citrate synthase OS=Homo sapiens OX=9606 GN=ACLY PE=1 SV=3
A0A087WWU8_HUMAN	TPM3	Tropomyosin alpha-3 chain OS=Homo sapiens OX=9606 GN=TPM3 PE=1 SV=1
P28074|PSB5_HUMAN	PSMB5	Proteasome subunit beta type-5 OS=Homo sapiens OX=9606 GN=PSMB5 PE=1 SV=3

Q86W42|THOC6_HUMAN	THOC6	THO complex subunit 6 homolog OS=Homo sapiens OX=9606 GN=THOC6 PE=1 SV=1
P67870|CSK2B_HUMAN	CSNK2B	Casein kinase II subunit beta OS=Homo sapiens OX=9606 GN=CSNK2B PE=1 SV=1
O15047|SET1A_HUMAN	SETD1A	Histone-lysine N-methyltransferase SETD1A OS=Homo sapiens OX=9606 GN=SETD1A PE=1 SV=3
Q9UQ13|SHOC2_HUMAN	SHOC2	Leucine-rich repeat protein SHOC-2 OS=Homo sapiens OX=9606 GN=SHOC2 PE=1 SV=2
P48426|PI42A_HUMAN	PIP4K2A	Phosphatidylinositol 5-phosphate 4-kinase type-2 alpha OS=Homo sapiens OX=9606 GN=PIP4K2A PE=1 SV=2
P61088|UBE2N_HUMAN	UBE2N	Ubiquitin-conjugating enzyme E2 N OS=Homo sapiens OX=9606 GN=UBE2N PE=1 SV=1
A0A6Q8PFE6_HUMAN	PTBP2	Polypyrimidine tract-binding protein 2 OS=Homo sapiens OX=9606 GN=PTBP2 PE=1 SV=1
O75475|PSIP1_HUMAN	PSIP1	PC4 and SFRS1-interacting protein OS=Homo sapiens OX=9606 GN=PSIP1 PE=1 SV=1
P78527|PRKDC_HUMAN	PRKDC	DNA-dependent protein kinase catalytic subunit OS=Homo sapiens OX=9606 GN=PRKDC PE=1 SV=3
P40227|TCPZ_HUMAN	CCT6A	T-complex protein 1 subunit zeta OS=Homo sapiens OX=9606 GN=CCT6A PE=1 SV=3
A0A3B3IUA7_HUMAN	TRIM25	E3 ubiquitin/ISG15 ligase TRIM25 OS=Homo sapiens OX=9606 GN=TRIM25 PE=1 SV=1
P50990|TCPQ_HUMAN	CCT8	T-complex protein 1 subunit theta OS=Homo sapiens OX=9606 GN=CCT8 PE=1 SV=4
P20962|PTMS_HUMAN	PTMS	Parathymosin OS=Homo sapiens OX=9606 GN=PTMS PE=1 SV=2
P48382|RFX5_HUMAN	RFX5	DNA-binding protein RFX5 OS=Homo sapiens OX=9606 GN=RFX5 PE=1 SV=1
tr|B9A041|B9A041_HUMAN	MDH1	Malate dehydrogenase cytoplasmic OS=Homo sapiens OX=9606 GN=MDH1 PE=1 SV=1
Q9UPN6|SCAF8_HUMAN	SCAF8	SR-related and CTD-associated factor 8 OS=Homo sapiens OX=9606 GN=SCAF8 PE=1 SV=1
tr|B8ZZL8|B8ZZL8_HUMAN	HSPE1	10 kDa heat shock protein mitochondrial OS=Homo sapiens OX=9606 GN=HSPE1 PE=1 SV=1
A0A3F2YNW7_HUMAN	ARID1B	AT-rich interactive domain-containing protein 1B OS=Homo sapiens OX=9606 GN=ARID1B PE=1 SV=1
P31942|HNRH3_HUMAN	HNRNPH3	Heterogeneous nuclear ribonucleoprotein H3 OS=Homo sapiens OX=9606 GN=HNRNPH3 PE=1 SV=2
tr|E5RK69|E5RK69_HUMAN	ANXA6	Annexin OS=Homo sapiens OX=9606 GN=ANXA6 PE=1 SV=1
P09496|CLCA_HUMAN	CLTA	Clathrin light chain A OS=Homo sapiens OX=9606 GN=CLTA PE=1 SV=1
Q8IU81|I2BP1_HUMAN	IRF2BP1	Interferon regulatory factor 2-binding protein 1 OS=Homo sapiens OX=9606 GN=IRF2BP1 PE=1 SV=1
tr|F2Z2T2|F2Z2T2_HUMAN	XPA	DNA repair protein-complementing XP-A cells OS=Homo sapiens OX=9606 GN=XPA PE=1 SV=1
tr|E7ET15|E7ET15_HUMAN	U2SURP	U2 snRNP-associated SURP motif-containing protein OS=Homo sapiens OX=9606 GN=U2SURP PE=1 SV=1
Q68EM7|RHG17_HUMAN	ARHGAP17	Rho GTPase-activating protein 17 OS=Homo sapiens OX=9606 GN=ARHGAP17 PE=1 SV=1
Q562R1|ACTBL_HUMAN	ACTBL2	Beta-actin-like protein 2 OS=Homo sapiens OX=9606 GN=ACTBL2 PE=1 SV=2
tr|S4R3E9|S4R3E9_HUMAN	NEDD8-MDP1	NEDD8 OS=Homo sapiens OX=9606 GN=NEDD8-MDP1 PE=3 SV=1
A0A669KB29_HUMAN	CXXC1	CXXC-type zinc finger protein 1 OS=Homo sapiens OX=9606 GN=CXXC1 PE=1 SV=1
tr|G8JLG1|G8JLG1_HUMAN	SMC1A	Structural maintenance of chromosomes protein OS=Homo sapiens OX=9606 GN=SMC1A PE=1 SV=2
Q9GZS3|WDR61_HUMAN	WDR61	WD repeat-containing protein 61 OS=Homo sapiens OX=9606 GN=WDR61 PE=1 SV=1
P05455|LA_HUMAN	SSB	Lupus La protein OS=Homo sapiens OX=9606 GN=SSB PE=1 SV=2
A0A7I2YQ95_HUMAN	KPNA4	Importin subunit alpha OS=Homo sapiens OX=9606 GN=KPNA4 PE=1 SV=1
O15014|ZN609_HUMAN	ZNF609	Zinc finger protein 609 OS=Homo sapiens OX=9606 GN=ZNF609 PE=1 SV=2
Q13151|ROA0_HUMAN	HNRNPA0	Heterogeneous nuclear ribonucleoprotein A0 OS=Homo sapiens OX=9606 GN=HNRNPA0 PE=1 SV=1

P49792|RBP2_HUMAN	RANBP2	E3 SUMO-protein ligase RanBP2 OS=Homo sapiens OX=9606 GN=RANBP2 PE=1 SV=2
A0A7P0T917_HUMAN	HSP90B1	Endoplasmin OS=Homo sapiens OX=9606 GN=HSP90B1 PE=1 SV=1
Q9UQE7|SMC3_HUMAN	SMC3	Structural maintenance of chromosomes protein 3 OS=Homo sapiens OX=9606 GN=SMC3 PE=1 SV=2
Q05048|CSTF1_HUMAN	CSTF1	Cleavage stimulation factor subunit 1 OS=Homo sapiens OX=9606 GN=CSTF1 PE=1 SV=1
O60231|DHX16_HUMAN	DHX16	Pre-mRNA-splicing factor ATP-dependent RNA helicase DHX16 OS=Homo sapiens OX=9606 GN=DHX16 PE=1 SV=2
O75146|HIP1R_HUMAN	HIP1R	Huntingtin-interacting protein 1-related protein OS=Homo sapiens OX=9606 GN=HIP1R PE=1 SV=2
P08579|RU2B_HUMAN	SNRPB2	U2 small nuclear ribonucleoprotein B'' OS=Homo sapiens OX=9606 GN=SNRPB2 PE=1 SV=1
tr|K7EL50|K7EL50_HUMAN	CALR	Calreticulin OS=Homo sapiens OX=9606 GN=CALR PE=1 SV=2
Q96BD5|PF21A_HUMAN	PHF21A	PHD finger protein 21A OS=Homo sapiens OX=9606 GN=PHF21A PE=1 SV=1
P60520|GBRL2_HUMAN	GABARAPL2	Gamma-aminobutyric acid receptor-associated protein-like 2 OS=Homo sapiens OX=9606 GN=GABARAPL2 PE=1 SV=1
P61962|DCAF7_HUMAN	DCAF7	DDB1- and CUL4-associated factor 7 OS=Homo sapiens OX=9606 GN=DCAF7 PE=1 SV=1
Q7L7X3|TAOK1_HUMAN	TAOK1	Serine/threonine-protein kinase TAO1 OS=Homo sapiens OX=9606 GN=TAOK1 PE=1 SV=1
P13051|UNG_HUMAN	UNG	Uracil-DNA glycosylase OS=Homo sapiens OX=9606 GN=UNG PE=1 SV=2
Q9NQG5|RPR1B_HUMAN	RPRD1B	Regulation of nuclear pre-mRNA domain-containing protein 1B OS=Homo sapiens OX=9606 GN=RPRD1B PE=1 SV=1
P37802|TAGL2_HUMAN	TAGLN2	Transgelin-2 OS=Homo sapiens OX=9606 GN=TAGLN2 PE=1 SV=3
Q9H2K8|TAOK3_HUMAN	TAOK3	Serine/threonine-protein kinase TAO3 OS=Homo sapiens OX=9606 GN=TAOK3 PE=1 SV=2
O43314|VIP2_HUMAN	PPIP5K2	Inositol hexakisphosphate and diphosphoinositol-pentakisphosphate kinase 2 OS=Homo sapiens OX=9606 GN=PPIP5K2 P
O14777|NDC80_HUMAN	NDC80	Kinetochore protein NDC80 homolog OS=Homo sapiens OX=9606 GN=NDC80 PE=1 SV=1
Q8TF74|WIPF2_HUMAN	WIPF2	WAS/WASL-interacting protein family member 2 OS=Homo sapiens OX=9606 GN=WIPF2 PE=1 SV=1
A0A7I2V3E1_HUMAN	PARP1	Poly [ADP-ribose] polymerase OS=Homo sapiens OX=9606 GN=PARP1 PE=1 SV=1
O60885|BRD4_HUMAN	BRD4	Bromodomain-containing protein 4 OS=Homo sapiens OX=9606 GN=BRD4 PE=1 SV=2
Q69YN4|VIR_HUMAN	VIRMA	Protein virilizer homolog OS=Homo sapiens OX=9606 GN=VIRMA PE=1 SV=2
tr|B1AKP7|B1AKP7_HUMAN	TARDBP	TAR DNA-binding protein 43 OS=Homo sapiens OX=9606 GN=TARDBP PE=1 SV=1
P34932|HSP74_HUMAN	HSPA4	Heat shock 70 kDa protein 4 OS=Homo sapiens OX=9606 GN=HSPA4 PE=1 SV=4
tr|G3V1C3|G3V1C3_HUMAN	API5	Apoptosis inhibitor 5 OS=Homo sapiens OX=9606 GN=API5 PE=1 SV=1
tr|G5E9D5|G5E9D5_HUMAN	ELAC2	ElaC homolog protein 2 OS=Homo sapiens OX=9606 GN=ELAC2 PE=1 SV=1
tr|F8VXI7|F8VXI7_HUMAN	DYNLL1	Dynein light chain (Fragment) OS=Homo sapiens OX=9606 GN=DYNLL1 PE=1 SV=1
Q12800|TFCP2_HUMAN	TFCP2	Alpha-globin transcription factor CP2 OS=Homo sapiens OX=9606 GN=TFCP2 PE=1 SV=2
Q8WWM7|ATX2L_HUMAN	ATXN2L	Ataxin-2-like protein OS=Homo sapiens OX=9606 GN=ATXN2L PE=1 SV=2
O00151|PDLI1_HUMAN	PDLIM1	PDZ and LIM domain protein 1 OS=Homo sapiens OX=9606 GN=PDLIM1 PE=1 SV=4
O00178|GTPB1_HUMAN	GTPBP1	GTP-binding protein 1 OS=Homo sapiens OX=9606 GN=GTPBP1 PE=1 SV=3
P62993|GRB2_HUMAN	GRB2	Growth factor receptor-bound protein 2 OS=Homo sapiens OX=9606 GN=GRB2 PE=1 SV=1
Q8NBT2|SPC24_HUMAN	SPC24	Kinetochore protein Spc24 OS=Homo sapiens OX=9606 GN=SPC24 PE=1 SV=2
Q9Y6I4|UBP3_HUMAN	USP3	Ubiquitin carboxyl-terminal hydrolase 3 OS=Homo sapiens OX=9606 GN=USP3 PE=1 SV=2

tr|F5GXF5|F5GXF5_HUMAN	BPTF	Nucleosome-remodeling factor subunit BPTF (Fragment) OS=Homo sapiens OX=9606 GN=BPTF PE=1 SV=2
Q8NE71|ABCF1_HUMAN	ABCF1	ATP-binding cassette sub-family F member 1 OS=Homo sapiens OX=9606 GN=ABCF1 PE=1 SV=2
tr|H3BVD1|H3BVD1_HUMAN	SMAD3	Mothers against decapentaplegic homolog (Fragment) OS=Homo sapiens OX=9606 GN=SMAD3 PE=1 SV=1
tr|B7Z5N5|B7Z5N5_HUMAN	SMAD2	Mothers against decapentaplegic homolog OS=Homo sapiens OX=9606 GN=SMAD2 PE=1 SV=1
O15198|SMAD9_HUMAN	SMAD9	Mothers against decapentaplegic homolog 9 OS=Homo sapiens OX=9606 GN=SMAD9 PE=1 SV=1
A0A024QZP7_HUMAN	CDK1	Cell division cycle 2 G1 to S and G2 to M isoform CRA_a OS=Homo sapiens OX=9606 GN=CDK1 PE=1 SV=1
O60264|SMCA5_HUMAN	SMARCA5	SWI/SNF-related matrix-associated actin-dependent regulator of chromatin subfamily A member 5 OS=Homo sapiens OX
A0A669KB12_HUMAN	EP300	Histone acetyltransferase OS=Homo sapiens OX=9606 GN=EP300 PE=1 SV=1
Q9BR76|COR1B_HUMAN	CORO1B	Coronin-1B OS=Homo sapiens OX=9606 GN=CORO1B PE=1 SV=1
Q15084|PDIA6_HUMAN	PDIA6	Protein disulfide-isomerase A6 OS=Homo sapiens OX=9606 GN=PDIA6 PE=1 SV=1
O14776|TCRG1_HUMAN	TCERG1	Transcription elongation regulator 1 OS=Homo sapiens OX=9606 GN=TCERG1 PE=1 SV=2
A0A087X0P4_HUMAN	PBX1	Pre-B-cell leukemia transcription factor 1 OS=Homo sapiens OX=9606 GN=PBX1 PE=1 SV=1
Q5MNZ6|WIPI3_HUMAN	WDR45B	WD repeat domain phosphoinositide-interacting protein 3 OS=Homo sapiens OX=9606 GN=WDR45B PE=1 SV=2
Q92733|PRCC_HUMAN	PRCC	Proline-rich protein PRCC OS=Homo sapiens OX=9606 GN=PRCC PE=1 SV=1
A0A087X271_HUMAN	CNN2	Calponin (Fragment) OS=Homo sapiens OX=9606 GN=CNN2 PE=1 SV=1
P31150|GDIA_HUMAN	GDI1	Rab GDP dissociation inhibitor alpha OS=Homo sapiens OX=9606 GN=GDI1 PE=1 SV=2
A0A7P0TAK7_HUMAN	DDB1	DNA damage-binding protein 1 OS=Homo sapiens OX=9606 GN=DDB1 PE=1 SV=1
tr|M0QXD6|M0QXD6_HUMAN	GTF2F1	Transcription initiation factor IIF subunit alpha (Fragment) OS=Homo sapiens OX=9606 GN=GTF2F1 PE=1 SV=1
P62633|CNBP_HUMAN	CNBP	CCHC-type zinc finger nucleic acid binding protein OS=Homo sapiens OX=9606 GN=CNBP PE=1 SV=1
Q9BY42|RTF2_HUMAN	RTF2	Replication termination factor 2 OS=Homo sapiens OX=9606 GN=RTF2 PE=1 SV=3
P78371|TCPB_HUMAN	CCT2	T-complex protein 1 subunit beta OS=Homo sapiens OX=9606 GN=CCT2 PE=1 SV=4
M0QY97_HUMAN	ZC3H4	Zinc finger CCCH domain-containing protein 4 (Fragment) OS=Homo sapiens OX=9606 GN=ZC3H4 PE=1 SV=1
P62136|PP1A_HUMAN	PPP1CA	Serine/threonine-protein phosphatase PP1-alpha catalytic subunit OS=Homo sapiens OX=9606 GN=PPP1CA PE=1 SV=1
tr|H0Y390|H0Y390_HUMAN	MACF1	Microtubule-actin cross-linking factor 1 isoforms 1/2/3/5 (Fragment) OS=Homo sapiens OX=9606 GN=MACF1 PE=1 SV=1
A0A6Q8PFX2_HUMAN	YARS1	Tyrosine--tRNA ligase OS=Homo sapiens OX=9606 GN=YARS1 PE=1 SV=1
B7ZBM3_HUMAN	FOXP4	Forkhead box protein P4 OS=Homo sapiens OX=9606 GN=FOXP4 PE=1 SV=1
O43929|ORC4_HUMAN	ORC4	Origin recognition complex subunit 4 OS=Homo sapiens OX=9606 GN=ORC4 PE=1 SV=2
C9JUJ0_HUMAN	CGGBP1	CGG triplet repeat-binding protein 1 (Fragment) OS=Homo sapiens OX=9606 GN=CGGBP1 PE=1 SV=1
H0YN26_HUMAN	ANP32A	Acidic leucine-rich nuclear phosphoprotein 32 family member A OS=Homo sapiens OX=9606 GN=ANP32A PE=1 SV=1
Q9BWJ5|SF3B5_HUMAN	SF3B5	Splicing factor 3B subunit 5 OS=Homo sapiens OX=9606 GN=SF3B5 PE=1 SV=1
Q12792|TWF1_HUMAN	TWF1	Twinfilin-1 OS=Homo sapiens OX=9606 GN=TWF1 PE=1 SV=3
Q96EK9|KTI12_HUMAN	KTI12	Protein KTI12 homolog OS=Homo sapiens OX=9606 GN=KTI12 PE=1 SV=1
F8VZJ2_HUMAN	NACA	Nascent polypeptide-associated complex subunit alpha OS=Homo sapiens OX=9606 GN=NACA PE=1 SV=1
H0Y2Q1_HUMAN	TRMT61A	tRNA (adenine(58)-N(1))-methyltransferase (Fragment) OS=Homo sapiens OX=9606 GN=TRMT61A PE=1 SV=1

P25789|PSA4_HUMAN	PSMA4	Proteasome subunit alpha type-4 OS=Homo sapiens OX=9606 GN=PSMA4 PE=1 SV=1
O14745|NHRF1_HUMAN	SLC9A3R1	Na(+)/H(+) exchange regulatory cofactor NHE-RF1 OS=Homo sapiens OX=9606 GN=SLC9A3R1 PE=1 SV=4
tr|Q8N450|Q8N450_HUMAN	FSD1L	FSD1-like protein OS=Homo sapiens OX=9606 GN=FSD1L PE=1 SV=1
Q16204|CCDC6_HUMAN	CCDC6	Coiled-coil domain-containing protein 6 OS=Homo sapiens OX=9606 GN=CCDC6 PE=1 SV=2
Q01167|FOXK2_HUMAN	FOXK2	Forkhead box protein K2 OS=Homo sapiens OX=9606 GN=FOXK2 PE=1 SV=3
P35250|RFC2_HUMAN	RFC2	Replication factor C subunit 2 OS=Homo sapiens OX=9606 GN=RFC2 PE=1 SV=3
tr|H0Y5T1|H0Y5T1_HUMAN	CLASP1	CLIP-associating protein 1 (Fragment) OS=Homo sapiens OX=9606 GN=CLASP1 PE=1 SV=2
Q99436|PSB7_HUMAN	PSMB7	Proteasome subunit beta type-7 OS=Homo sapiens OX=9606 GN=PSMB7 PE=1 SV=1
Q9H7L9|SDS3_HUMAN	SUDS3	Sin3 histone deacetylase corepressor complex component SDS3 OS=Homo sapiens OX=9606 GN=SUDS3 PE=1 SV=2
Q9Y2S6|TMA7_HUMAN	TMA7	Translation machinery-associated protein 7 OS=Homo sapiens OX=9606 GN=TMA7 PE=1 SV=1
P25205|MCM3_HUMAN	MCM3	DNA replication licensing factor MCM3 OS=Homo sapiens OX=9606 GN=MCM3 PE=1 SV=3
P46109|CRKL_HUMAN	CRKL	Crk-like protein OS=Homo sapiens OX=9606 GN=CRKL PE=1 SV=1
tr|J3KSH1|J3KSH1_HUMAN	AMZ2	Archaemetzincin-2 (Fragment) OS=Homo sapiens OX=9606 GN=AMZ2 PE=1 SV=1
tr|Q5TG40|Q5TG40_HUMAN	DMAP1	DNA methyltransferase 1-associated protein 1 (Fragment) OS=Homo sapiens OX=9606 GN=DMAP1 PE=1 SV=1
tr|G3V1J5|G3V1J5_HUMAN	DIS3	Exosome complex exonuclease RRP44 OS=Homo sapiens OX=9606 GN=DIS3 PE=1 SV=1
A0A0A0MSK6_HUMAN	ARHGAP5	Rho GTPase-activating protein 5 OS=Homo sapiens OX=9606 GN=ARHGAP5 PE=1 SV=1
tr|E9PJ06|E9PJ06_HUMAN	CSTF3	Cleavage stimulation factor subunit 3 (Fragment) OS=Homo sapiens OX=9606 GN=CSTF3 PE=1 SV=1
Q96CT7|CC124_HUMAN	CCDC124	Coiled-coil domain-containing protein 124 OS=Homo sapiens OX=9606 GN=CCDC124 PE=1 SV=1
Q14974|IMB1_HUMAN	KPNB1	Importin subunit beta-1 OS=Homo sapiens OX=9606 GN=KPNB1 PE=1 SV=2
P50991|TCPD_HUMAN	CCT4	T-complex protein 1 subunit delta OS=Homo sapiens OX=9606 GN=CCT4 PE=1 SV=4
Q9HB71|CYBP_HUMAN	CACYBP	Calcyclin-binding protein OS=Homo sapiens OX=9606 GN=CACYBP PE=1 SV=2
Q8TAF3|WDR48_HUMAN	WDR48	WD repeat-containing protein 48 OS=Homo sapiens OX=9606 GN=WDR48 PE=1 SV=1
Q6GQQ9|OTU7B_HUMAN	OTUD7B	OTU domain-containing protein 7B OS=Homo sapiens OX=9606 GN=OTUD7B PE=1 SV=1
tr|I3L3H2|I3L3H2_HUMAN	EIF4A3	RNA helicase OS=Homo sapiens OX=9606 GN=EIF4A3 PE=1 SV=2
tr|C9JE98|C9JE98_HUMAN	NCOR2	Nuclear receptor corepressor 2 OS=Homo sapiens OX=9606 GN=NCOR2 PE=1 SV=1
A0A7I2V3K7_HUMAN	DNAJB1	DnaJ homolog subfamily B member 1 OS=Homo sapiens OX=9606 GN=DNAJB1 PE=1 SV=1
Q8WW12|PCNP_HUMAN	PCNP	PEST proteolytic signal-containing nuclear protein OS=Homo sapiens OX=9606 GN=PCNP PE=1 SV=2
P14550|AK1A1_HUMAN	AKR1A1	Aldo-keto reductase family 1 member A1 OS=Homo sapiens OX=9606 GN=AKR1A1 PE=1 SV=3
Q8TDJ6|DMXL2_HUMAN	DMXL2	DmX-like protein 2 OS=Homo sapiens OX=9606 GN=DMXL2 PE=1 SV=2
Q8TBX8|PI42C_HUMAN	PIP4K2C	Phosphatidylinositol 5-phosphate 4-kinase type-2 gamma OS=Homo sapiens OX=9606 GN=PIP4K2C PE=1 SV=3
Q08J23|NSUN2_HUMAN	NSUN2	RNA cytosine C(5)-methyltransferase NSUN2 OS=Homo sapiens OX=9606 GN=NSUN2 PE=1 SV=2
A0A0U1RQR8_HUMAN	FOXP2	Forkhead box protein P2 OS=Homo sapiens OX=9606 GN=FOXP2 PE=1 SV=1
P49750|YLPM1_HUMAN	YLPM1	YLP motif-containing protein 1 OS=Homo sapiens OX=9606 GN=YLPM1 PE=1 SV=4
Q99962|SH3G2_HUMAN	SH3GL2	Endophilin-A1 OS=Homo sapiens OX=9606 GN=SH3GL2 PE=1 SV=1

tr|M0QYE0|M0QYE0_HUMAN	SH3GL1	Endophilin-A2 (Fragment) OS=Homo sapiens OX=9606 GN=SH3GL1 PE=1 SV=8
P55795|HNRH2_HUMAN	HNRNPH2	Heterogeneous nuclear ribonucleoprotein H2 OS=Homo sapiens OX=9606 GN=HNRNPH2 PE=1 SV=1
Q63HQ2|EGFLA_HUMAN	EGFLAM	Pikachurin OS=Homo sapiens OX=9606 GN=EGFLAM PE=1 SV=2
P17987|TCPA_HUMAN	TCP1	T-complex protein 1 subunit alpha OS=Homo sapiens OX=9606 GN=TCP1 PE=1 SV=1
tr|J3KTL8|J3KTL8_HUMAN	SMCHD1	Structural maintenance of chromosomes flexible hinge domain-containing protein 1 (Fragment) OS=Homo sapiens OX=9[illegible]
Q9Y3F4|STRAP_HUMAN	STRAP	Serine-threonine kinase receptor-associated protein OS=Homo sapiens OX=9606 GN=STRAP PE=1 SV=1
tr|H0YFG1|H0YFG1_HUMAN	EPS8	Epidermal growth factor receptor kinase substrate 8 (Fragment) OS=Homo sapiens OX=9606 GN=EPS8 PE=1 SV=1
P07814|SYEP_HUMAN	EPRS1	Bifunctional glutamate/proline--tRNA ligase OS=Homo sapiens OX=9606 GN=EPRS1 PE=1 SV=5
Q9NQT5|EXOS3_HUMAN	EXOSC3	Exosome complex component RRP40 OS=Homo sapiens OX=9606 GN=EXOSC3 PE=1 SV=3
Q16643|DREB_HUMAN	DBN1	Drebrin OS=Homo sapiens OX=9606 GN=DBN1 PE=1 SV=4
P05198|IF2A_HUMAN	EIF2S1	Eukaryotic translation initiation factor 2 subunit 1 OS=Homo sapiens OX=9606 GN=EIF2S1 PE=1 SV=3
Q99832|TCPH_HUMAN	CCT7	T-complex protein 1 subunit eta OS=Homo sapiens OX=9606 GN=CCT7 PE=1 SV=2
tr|H0YF33|H0YF33_HUMAN	NASP	Nuclear autoantigenic sperm protein (Fragment) OS=Homo sapiens OX=9606 GN=NASP PE=1 SV=1
Q96L91|EP400_HUMAN	EP400	E1A-binding protein p400 OS=Homo sapiens OX=9606 GN=EP400 PE=1 SV=4
A0A494C0S2_HUMAN	ERCC5	DNA excision repair protein ERCC-5 OS=Homo sapiens OX=9606 GN=ERCC5 PE=1 SV=1
A0A1W2PS85_HUMAN	BIVM-ERCC5	BIVM-ERCC5 readthrough OS=Homo sapiens OX=9606 GN=BIVM-ERCC5 PE=1 SV=1
Q92618|ZN516_HUMAN	ZNF516	Zinc finger protein 516 OS=Homo sapiens OX=9606 GN=ZNF516 PE=1 SV=1
Q86YP4|P66A_HUMAN	GATAD2A	Transcriptional repressor p66-alpha OS=Homo sapiens OX=9606 GN=GATAD2A PE=1 SV=1
tr|C9J4Q3|C9J4Q3_HUMAN	PALS2	Protein PALS2 (Fragment) OS=Homo sapiens OX=9606 GN=PALS2 PE=1 SV=1
P26640|SYVC_HUMAN	VARS1	Valine--tRNA ligase OS=Homo sapiens OX=9606 GN=VARS1 PE=1 SV=4
Q14195|DPYL3_HUMAN	DPYSL3	Dihydropyrimidinase-related protein 3 OS=Homo sapiens OX=9606 GN=DPYSL3 PE=1 SV=1
P49368|TCPG_HUMAN	CCT3	T-complex protein 1 subunit gamma OS=Homo sapiens OX=9606 GN=CCT3 PE=1 SV=4
Q9NS91|RAD18_HUMAN	RAD18	E3 ubiquitin-protein ligase RAD18 OS=Homo sapiens OX=9606 GN=RAD18 PE=1 SV=2
A0A024RA52_HUMAN	PSMA2	Proteasome subunit alpha type OS=Homo sapiens OX=9606 GN=PSMA2 PE=1 SV=1
Q9BW19|KIFC1_HUMAN	KIFC1	Kinesin-like protein KIFC1 OS=Homo sapiens OX=9606 GN=KIFC1 PE=1 SV=2
tr|B1APG0|B1APG0_HUMAN	PRKACB	cAMP-dependent protein kinase catalytic subunit beta (Fragment) OS=Homo sapiens OX=9606 GN=PRKACB PE=1 SV=1
tr|E2QRF9|E2QRF9_HUMAN	GMNN	Geminin (Fragment) OS=Homo sapiens OX=9606 GN=GMNN PE=1 SV=1
F5GWN1_HUMAN	SS18	Protein SSXT OS=Homo sapiens OX=9606 GN=SS18 PE=1 SV=2
A0A1W2PRB5_HUMAN	KANSL1	KAT8 regulatory NSL complex subunit 1 OS=Homo sapiens OX=9606 GN=KANSL1 PE=1 SV=1
tr|Q9NW25|Q9NW25_HUMAN	SSBP3	Single stranded DNA binding protein 3 isoform CRA_c OS=Homo sapiens OX=9606 GN=SSBP3 PE=1 SV=1
Q96L92|SNX27_HUMAN	SNX27	Sorting nexin-27 OS=Homo sapiens OX=9606 GN=SNX27 PE=1 SV=2
tr|F8WBL2|F8WBL2_HUMAN	BYSL	Bystin OS=Homo sapiens OX=9606 GN=BYSL PE=1 SV=1
tr|H0YBX6|H0YBX6_HUMAN	UBE2V2	Ubiquitin-conjugating enzyme E2 variant 2 (Fragment) OS=Homo sapiens OX=9606 GN=UBE2V2 PE=1 SV=1
tr|D6RG00|D6RG00_HUMAN	UBE2V1	HCG2018358 isoform CRA_d OS=Homo sapiens OX=9606 GN=UBE2V1 PE=4 SV=2

tr|I3L0A0|I3L0A0_HUMAN	PEDS1-UBE2V1	HCG2044781 OS=Homo sapiens OX=9606 GN=PEDS1-UBE2V1 PE=1 SV=1
Q9Y6M1|IF2B2_HUMAN	IGF2BP2	Insulin-like growth factor 2 mRNA-binding protein 2 OS=Homo sapiens OX=9606 GN=IGF2BP2 PE=1 SV=2
Q99733|NP1L4_HUMAN	NAP1L4	Nucleosome assembly protein 1-like 4 OS=Homo sapiens OX=9606 GN=NAP1L4 PE=1 SV=1
A0A2R8Y7M3_HUMAN	RDX	Radixin OS=Homo sapiens OX=9606 GN=RDX PE=1 SV=1
Q15906|VPS72_HUMAN	VPS72	Vacuolar protein sorting-associated protein 72 homolog OS=Homo sapiens OX=9606 GN=VPS72 PE=1 SV=1
A0A7P0TA47_HUMAN	DLAT	Acetyltransferase component of pyruvate dehydrogenase complex OS=Homo sapiens OX=9606 GN=DLAT PE=1 SV=1
tr|E9PHT9|E9PHT9_HUMAN	ANXA5	Annexin OS=Homo sapiens OX=9606 GN=ANXA5 PE=1 SV=1
tr|B7Z4S1|B7Z4S1_HUMAN	TCEA1	Transcription elongation factor A protein 1 OS=Homo sapiens OX=9606 GN=TCEA1 PE=1 SV=1
tr|Q5JRI7|Q5JRI7_HUMAN	TCEA2	Transcription elongation factor A protein 2 (Fragment) OS=Homo sapiens OX=9606 GN=TCEA2 PE=1 SV=1
tr|G3V4R3|G3V4R3_HUMAN	PPP4R3A	Serine/threonine-protein phosphatase 4 regulatory subunit 3A (Fragment) OS=Homo sapiens OX=9606 GN=PPP4R3A PE=
Q5MIZ7|P4R3B_HUMAN	PPP4R3B	Serine/threonine-protein phosphatase 4 regulatory subunit 3B OS=Homo sapiens OX=9606 GN=PPP4R3B PE=1 SV=2
tr|H0YJ92|H0YJ92_HUMAN	MNAT1	CDK-activating kinase assembly factor MAT1 (Fragment) OS=Homo sapiens OX=9606 GN=MNAT1 PE=1 SV=1
Q96D46|NMD3_HUMAN	NMD3	60S ribosomal export protein NMD3 OS=Homo sapiens OX=9606 GN=NMD3 PE=1 SV=1
Q16555|DPYL2_HUMAN	DPYSL2	Dihydropyrimidinase-related protein 2 OS=Homo sapiens OX=9606 GN=DPYSL2 PE=1 SV=1
Q92729|PTPRU_HUMAN	PTPRU	Receptor-type tyrosine-protein phosphatase U OS=Homo sapiens OX=9606 GN=PTPRU PE=1 SV=2
P25098|ARBK1_HUMAN	GRK2	Beta-adrenergic receptor kinase 1 OS=Homo sapiens OX=9606 GN=GRK2 PE=1 SV=2
P35626|ARBK2_HUMAN	GRK3	Beta-adrenergic receptor kinase 2 OS=Homo sapiens OX=9606 GN=GRK3 PE=1 SV=2
Q96BZ8|LENG1_HUMAN	LENG1	Leukocyte receptor cluster member 1 OS=Homo sapiens OX=9606 GN=LENG1 PE=1 SV=1
Q9NW82|WDR70_HUMAN	WDR70	WD repeat-containing protein 70 OS=Homo sapiens OX=9606 GN=WDR70 PE=1 SV=1
Q01538|MYT1_HUMAN	MYT1	Myelin transcription factor 1 OS=Homo sapiens OX=9606 GN=MYT1 PE=1 SV=2
A0A3B3IU83_HUMAN	MYT1L	Myelin transcription factor 1-like protein (Fragment) OS=Homo sapiens OX=9606 GN=MYT1L PE=1 SV=1
tr|E5RHS3|E5RHS3_HUMAN	ST18	Suppression of tumorigenicity 18 protein (Fragment) OS=Homo sapiens OX=9606 GN=ST18 PE=1 SV=1
A0A087WYF8_HUMAN	PDLIM3	PDZ and LIM domain protein 3 OS=Homo sapiens OX=9606 GN=PDLIM3 PE=1 SV=2
P04637|P53_HUMAN	TP53	Cellular tumor antigen p53 OS=Homo sapiens OX=9606 GN=TP53 PE=1 SV=4
A0A7I2V4X1_HUMAN	SETBP1	SET-binding protein OS=Homo sapiens OX=9606 GN=SETBP1 PE=1 SV=1
tr|B1ALK7|B1ALK7_HUMAN	ARHGEF7	Rho guanine nucleotide exchange factor 7 OS=Homo sapiens OX=9606 GN=ARHGEF7 PE=1 SV=1
Q96CT2|KLH29_HUMAN	KLHL29	Kelch-like protein 29 OS=Homo sapiens OX=9606 GN=KLHL29 PE=1 SV=3
tr|E9PD53|E9PD53_HUMAN	SMC4	Structural maintenance of chromosomes protein OS=Homo sapiens OX=9606 GN=SMC4 PE=1 SV=1
tr|B9ZVV8|B9ZVV8_HUMAN	SETMAR	Histone-lysine N-methyltransferase SETMAR (Fragment) OS=Homo sapiens OX=9606 GN=SETMAR PE=1 SV=2
A0A7I2YQK6_HUMAN	HSPD1	60 kDa chaperonin OS=Homo sapiens OX=9606 GN=HSPD1 PE=1 SV=1
P63208|SKP1_HUMAN	SKP1	S-phase kinase-associated protein 1 OS=Homo sapiens OX=9606 GN=SKP1 PE=1 SV=2
tr|F5H497|F5H497_HUMAN	KCTD10	BTB/POZ domain-containing adapter for CUL3-mediated RhoA degradation protein 3 (Fragment) OS=Homo sapiens OX=9
Q96BY6|DOC10_HUMAN	DOCK10	Dedicator of cytokinesis protein 10 OS=Homo sapiens OX=9606 GN=DOCK10 PE=1 SV=3
O95400|CD2B2_HUMAN	CD2BP2	CD2 antigen cytoplasmic tail-binding protein 2 OS=Homo sapiens OX=9606 GN=CD2BP2 PE=1 SV=1

A0A6Q8PGR4_HUMAN	CEP41	Centrosomal protein of 41 kDa OS=Homo sapiens OX=9606 GN=CEP41 PE=1 SV=1
tr|B9EGQ5|B9EGQ5_HUMAN	WIZ	Protein Wiz OS=Homo sapiens OX=9606 GN=WIZ PE=1 SV=1
Q29RF7|PDS5A_HUMAN	PDS5A	Sister chromatid cohesion protein PDS5 homolog A OS=Homo sapiens OX=9606 GN=PDS5A PE=1 SV=1
Q16658|FSCN1_HUMAN	FSCN1	Fascin OS=Homo sapiens OX=9606 GN=FSCN1 PE=1 SV=3
O15037|KHNYN_HUMAN	KHNYN	Protein KHNYN OS=Homo sapiens OX=9606 GN=KHNYN PE=1 SV=3
A0A7P0TB08_HUMAN	VRK1	Serine/threonine-protein kinase VRK1 OS=Homo sapiens OX=9606 GN=VRK1 PE=1 SV=1
tr|E9PLG2|E9PLG2_HUMAN	PSMC3	26S proteasome regulatory subunit 6A (Fragment) OS=Homo sapiens OX=9606 GN=PSMC3 PE=1 SV=3
Q9Y6X9|MORC2_HUMAN	MORC2	ATPase MORC2 OS=Homo sapiens OX=9606 GN=MORC2 PE=1 SV=2
A0A075B6Q2_HUMAN	FNBP1L	Formin-binding protein 1-like (Fragment) OS=Homo sapiens OX=9606 GN=FNBP1L PE=1 SV=1
tr|C9J4K5|C9J4K5_HUMAN	DONSON	Protein downstream neighbor of Son OS=Homo sapiens OX=9606 GN=DONSON PE=1 SV=1
tr|F5GYS8|F5GYS8_HUMAN	MEIS1	Homeobox protein Meis1 OS=Homo sapiens OX=9606 GN=MEIS1 PE=1 SV=1
Q9NXV6|CARF_HUMAN	CDKN2AIP	CDKN2A-interacting protein OS=Homo sapiens OX=9606 GN=CDKN2AIP PE=1 SV=3
O75937|DNJC8_HUMAN	DNAJC8	DnaJ homolog subfamily C member 8 OS=Homo sapiens OX=9606 GN=DNAJC8 PE=1 SV=2
Q9BVA1|TBB2B_HUMAN	TUBB2B	Tubulin beta-2B chain OS=Homo sapiens OX=9606 GN=TUBB2B PE=1 SV=1
Q99592|ZBT18_HUMAN	ZBTB18	Zinc finger and BTB domain-containing protein 18 OS=Homo sapiens OX=9606 GN=ZBTB18 PE=1 SV=1
Q12789|TF3C1_HUMAN	GTF3C1	General transcription factor 3C polypeptide 1 OS=Homo sapiens OX=9606 GN=GTF3C1 PE=1 SV=4
A0A087WZG9_HUMAN	PEG10	Retrotransposon-derived protein PEG10 OS=Homo sapiens OX=9606 GN=PEG10 PE=1 SV=2
Q92917|GPKOW_HUMAN	GPKOW	G-patch domain and KOW motifs-containing protein OS=Homo sapiens OX=9606 GN=GPKOW PE=1 SV=2
Q9C0B0|UNK_HUMAN	UNK	RING finger protein unkempt homolog OS=Homo sapiens OX=9606 GN=UNK PE=1 SV=2
tr|R4GMN4|R4GMN4_HUMAN	THOC1	THO complex subunit 1 (Fragment) OS=Homo sapiens OX=9606 GN=THOC1 PE=1 SV=1
Q9BWU1|CDK19_HUMAN	CDK19	Cyclin-dependent kinase 19 OS=Homo sapiens OX=9606 GN=CDK19 PE=1 SV=1
Q7Z2T5|TRM1L_HUMAN	TRMT1L	TRMT1-like protein OS=Homo sapiens OX=9606 GN=TRMT1L PE=1 SV=2
tr|C9J0J7|C9J0J7_HUMAN	PFN2	Profilin OS=Homo sapiens OX=9606 GN=PFN2 PE=1 SV=1
Q10570|CPSF1_HUMAN	CPSF1	Cleavage and polyadenylation specificity factor subunit 1 OS=Homo sapiens OX=9606 GN=CPSF1 PE=1 SV=2
P46939|UTRN_HUMAN	UTRN	Utrophin OS=Homo sapiens OX=9606 GN=UTRN PE=1 SV=2
Q96QC0|PP1RA_HUMAN	PPP1R10	Serine/threonine-protein phosphatase 1 regulatory subunit 10 OS=Homo sapiens OX=9606 GN=PPP1R10 PE=1 SV=1
tr|E7ESI2|E7ESI2_HUMAN	CDK2	Cyclin-dependent kinase 2 OS=Homo sapiens OX=9606 GN=CDK2 PE=1 SV=1
P13667|PDIA4_HUMAN	PDIA4	Protein disulfide-isomerase A4 OS=Homo sapiens OX=9606 GN=PDIA4 PE=1 SV=2
P13861|KAP2_HUMAN	PRKAR2A	cAMP-dependent protein kinase type II-alpha regulatory subunit OS=Homo sapiens OX=9606 GN=PRKAR2A PE=1 SV=2
Q92616|GCN1_HUMAN	GCN1	eIF-2-alpha kinase activator GCN1 OS=Homo sapiens OX=9606 GN=GCN1 PE=1 SV=6
O00401|WASL_HUMAN	WASL	Neural Wiskott-Aldrich syndrome protein OS=Homo sapiens OX=9606 GN=WASL PE=1 SV=2
tr|G3V500|G3V500_HUMAN	EML1	Echinoderm microtubule-associated protein-like 1 (Fragment) OS=Homo sapiens OX=9606 GN=EML1 PE=1 SV=1
tr|F5H365|F5H365_HUMAN	SEC23A	Protein transport protein SEC23 OS=Homo sapiens OX=9606 GN=SEC23A PE=1 SV=1
O15379|HDAC3_HUMAN	HDAC3	Histone deacetylase 3 OS=Homo sapiens OX=9606 GN=HDAC3 PE=1 SV=2

Q9NXC5|MIO_HUMAN	MIOS	GATOR complex protein MIOS OS=Homo sapiens OX=9606 GN=MIOS PE=1 SV=2
P29084|T2EB_HUMAN	GTF2E2	Transcription initiation factor IIE subunit beta OS=Homo sapiens OX=9606 GN=GTF2E2 PE=1 SV=1
A0A1B0GV09_HUMAN	EHMT1	Histone-lysine N-methyltransferase EHMT1 OS=Homo sapiens OX=9606 GN=EHMT1 PE=1 SV=1
O95551|TYDP2_HUMAN	TDP2	Tyrosyl-DNA phosphodiesterase 2 OS=Homo sapiens OX=9606 GN=TDP2 PE=1 SV=1
Q96B26|EXOS8_HUMAN	EXOSC8	Exosome complex component RRP43 OS=Homo sapiens OX=9606 GN=EXOSC8 PE=1 SV=1
A0A2R8Y611_HUMAN	AP3D1	AP-3 complex subunit delta (Fragment) OS=Homo sapiens OX=9606 GN=AP3D1 PE=1 SV=1
Q00341|VIGLN_HUMAN	HDLBP	Vigilin OS=Homo sapiens OX=9606 GN=HDLBP PE=1 SV=2
A0A804HI55_HUMAN	BCL9	B-cell CLL/lymphoma 9 protein OS=Homo sapiens OX=9606 GN=BCL9 PE=4 SV=1
Q96RS6|NUDC1_HUMAN	NUDCD1	NudC domain-containing protein 1 OS=Homo sapiens OX=9606 GN=NUDCD1 PE=1 SV=2
Q8IWX8|CHERP_HUMAN	CHERP	Calcium homeostasis endoplasmic reticulum protein OS=Homo sapiens OX=9606 GN=CHERP PE=1 SV=3
Q6STE5|SMRD3_HUMAN	SMARCD3	SWI/SNF-related matrix-associated actin-dependent regulator of chromatin subfamily D member 3 OS=Homo sapiens OX
tr|H0YGQ6|H0YGQ6_HUMAN	RFXANK	DNA-binding protein RFXANK (Fragment) OS=Homo sapiens OX=9606 GN=RFXANK PE=1 SV=1
A0A804CYY4_HUMAN	DPF1	D4 zinc and double PHD fingers family 1 isoform CRA_b OS=Homo sapiens OX=9606 GN=DPF1 PE=4 SV=1

506 GN=SMCHD1 PE=1 SV=1

)606 GN=KCTD10 PE=1 SV=1

=9606 GN=SMARCD3 PE=1 SV=1
