# Supplementary material for: Polypyrimidine tract binding proteins PTBP1 and PTBP2 associate with distinct proteins and have distinct post-translational modifications in neuronal nuclear extract
Source: PLoS One. 2025 Jun 4;20(6):e0325143. doi: 10.1371/journal.pone.0325143 (PMC12136456; doi:10.1371/journal.pone.0325143)
Supplement: S4 Table — Unique proteins that either directly or indirectly (via bound RNA) interact and co-elute with both PTBP1 and PTBP2 are listed in this table. Proteins identified as unspecifically bound to the Ni2 + beads and carried over during recombinant expression and purification were removed from this list. (PDF) [file pone.0325143.s007.pdf]

Sppl. Table. 4. Proteins that were common and present in both PTBP1 and PTBP2 eluates in WERI nuclear extract.

Protein Name	Gene Name	Description
F8WJN3_HUMAN	CPSF6	Cleavage and polyadenylation specificity factor subunit 6 OS=Homo sapiens OX=9606 GN=CPSF6 PE=1 SV=1
F5H669_HUMAN	CPSF7	Cleavage and polyadenylation-specificity factor subunit 7 (Fragment) OS=Homo sapiens OX=9606 GN=CPSF7 PE[illegible]
SETLP_HUMAN	SETSIP	Protein SETSIP OS=Homo sapiens OX=9606 GN=SETSIP PE=1 SV=1
J3QLE5_HUMAN	SNRPN	Small nuclear ribonucleoprotein-associated protein N (Fragment) OS=Homo sapiens OX=9606 GN=SNRPN PE=1 [illegible]
Q49AN9_HUMAN	SNRPG	Small nuclear ribonucleoprotein G OS=Homo sapiens OX=9606 GN=SNRPG PE=1 SV=1
F8W6I7_HUMAN	HNRNPA1	Helix-destabilizing protein OS=Homo sapiens OX=9606 GN=HNRNPA1 PE=1 SV=2
TDRD5_HUMAN	TDRD5	Tudor domain-containing protein 5 OS=Homo sapiens OX=9606 GN=TDRD5 PE=1 SV=3
HCFC1_HUMAN	HCFC1	Host cell factor 1 OS=Homo sapiens OX=9606 GN=HCFC1 PE=1 SV=2
A0A7I2V5M5_HUMAN	NCL	Nucleolin OS=Homo sapiens OX=9606 GN=NCL PE=1 SV=1
D6RFF0_HUMAN	LARP7	La ribonucleoprotein domain family member 7 (Fragment) OS=Homo sapiens OX=9606 GN=LARP7 PE=1 SV=1
K7EP06_HUMAN	RNMT	RG7MT1 OS=Homo sapiens OX=9606 GN=RNMT PE=1 SV=1
E7EX29_HUMAN	YWHAZ	14-3-3 protein zeta/delta (Fragment) OS=Homo sapiens OX=9606 GN=YWHAZ PE=1 SV=1
B0S8I6_HUMAN	FAM50A	Protein FAM50A (Fragment) OS=Homo sapiens OX=9606 GN=FAM50A PE=1 SV=1
H3BND8_HUMAN	USP7	Ubiquitin carboxyl-terminal hydrolase (Fragment) OS=Homo sapiens OX=9606 GN=USP7 PE=1 SV=1
Q4VY20_HUMAN	YWHAB	14-3-3 protein beta/alpha (Fragment) OS=Homo sapiens OX=9606 GN=YWHAB PE=1 SV=1

=1 SV=1

SV=1
