# Supplementary material for: Polypyrimidine tract binding proteins PTBP1 and PTBP2 associate with distinct proteins and have distinct post-translational modifications in neuronal nuclear extract
Source: PLoS One. 2025 Jun 4;20(6):e0325143. doi: 10.1371/journal.pone.0325143 (PMC12136456; doi:10.1371/journal.pone.0325143)
Supplement: S5 Table — Unique proteins that interact and co-elute with PTBP2 are listed in this table. Proteins identified as unspecifically bound to the Ni2 + beads and carried over during recombinant expression and purification have been removed from this list. (PDF) [file pone.0325143.s008.pdf]

Sppl. Table. 5. Proteins that co-purified and were unique to PTBP2 incubated in neuronal WERI nuclear extract

Accession (PTBI	Gene Name	Description
B4DWT1	SRSF11	Serine/arginine-rich-splicing factor 11 OS=Homo sapiens OX=9606 GN=SRSF11 PE=1 SV=1
A0A7I2V659	EEF1A1	Elongation factor 1-alpha OS=Homo sapiens OX=9606 GN=EEF1A1 PE=1 SV=1
Q96QS3	ARX	Homeobox protein ARX OS=Homo sapiens OX=9606 GN=ARX PE=1 SV=1
B7ZM99	MTHFD1L	Formyltetrahydrofolate synthetase OS=Homo sapiens OX=9606 GN=MTHFD1L PE=1 SV=1
B3KM87	MATR3	Matrin-3 OS=Homo sapiens OX=9606 GN=MATR3 PE=1 SV=1
B8ZZ98	SNRNP27	U4/U6.U5 small nuclear ribonucleoprotein 27 kDa protein OS=Homo sapiens OX=9606 GN=SNRNP27 PE=1 SV=1
A0A2R8Y765	SMARCE1	SWI/SNF-related matrix-associated actin-dependent regulator of chromatin subfamily E member 1 OS=Homo sapiens OX=9606 GN=S
G5E9W3	CPSF3	Cleavage and polyadenylation specific factor 3 73kDa isoform CRA_b OS=Homo sapiens OX=9606 GN=CPSF3 PE=1 SV=1
G3V570	MAX	Protein max (Fragment) OS=Homo sapiens OX=9606 GN=MAX PE=1 SV=8
F8WC91	USP39	U4/U6.U5 tri-snRNP-associated protein 2 OS=Homo sapiens OX=9606 GN=USP39 PE=1 SV=1
F8VXC8	SMARCC2	SWI/SNF complex subunit SMARCC2 OS=Homo sapiens OX=9606 GN=SMARCC2 PE=1 SV=1
A0A0D9SES8	TLE3	Transducin-like enhancer protein 3 OS=Homo sapiens OX=9606 GN=TLE3 PE=1 SV=1
A0A1W2PP22	HNRNPU	Heterogeneous nuclear ribonucleoprotein U (Fragment) OS=Homo sapiens OX=9606 GN=HNRNPU PE=1 SV=1
F5H3U9	MAGOHB	Protein mago nashi homolog 2 OS=Homo sapiens OX=9606 GN=MAGOHB PE=3 SV=1
P61326	MAGOH	Protein mago nashi homolog OS=Homo sapiens OX=9606 GN=MAGOH PE=1 SV=1
Q92688	ANP32B	Acidic leucine-rich nuclear phosphoprotein 32 family member B OS=Homo sapiens OX=9606 GN=ANP32B PE=1 SV=1
Q9NRR5	UBQLN4	Ubiquilin-4 OS=Homo sapiens OX=9606 GN=UBQLN4 PE=1 SV=2
Q9BRL6	SRSF8	Serine/arginine-rich splicing factor 8 OS=Homo sapiens OX=9606 GN=SRSF8 PE=1 SV=1
Q1XH10	SKIDA1	SKI/DACH domain-containing protein 1 OS=Homo sapiens OX=9606 GN=SKIDA1 PE=1 SV=2
J3QLN4	MIF4GD	MIF4G domain-containing protein OS=Homo sapiens OX=9606 GN=MIF4GD PE=1 SV=1
A0A7I2V2S0	DDX5	RNA helicase OS=Homo sapiens OX=9606 GN=DDX5 PE=1 SV=1
E7ERH2	SKP1	Cyclin-A/CDK2-associated protein p19 (Fragment) OS=Homo sapiens OX=9606 GN=SKP1 PE=1 SV=1
H0Y9L8	RIOX2	Bifunctional lysine-specific demethylase and histidyl-hydroxylase (Fragment) OS=Homo sapiens OX=9606 GN=RIOX2 PE=1 SV=1
E9PFI4	LDB2	LIM domain-binding protein 2 OS=Homo sapiens OX=9606 GN=LDB2 PE=1 SV=1
B8ZZ77	PPIL3	Peptidyl-prolyl cis-trans isomerase OS=Homo sapiens OX=9606 GN=PPIL3 PE=1 SV=1
F6VDE0	SMARCA2	Probable global transcription activator SNF2L2 OS=Homo sapiens OX=9606 GN=SMARCA2 PE=1 SV=2
H0YB39	HNRNPH1	Heterogeneous nuclear ribonucleoprotein H (Fragment) OS=Homo sapiens OX=9606 GN=HNRNPH1 PE=1 SV=1
A0A7I2YQQ0	DNMT1	DNA (cytosine-5)-methyltransferase OS=Homo sapiens OX=9606 GN=DNMT1 PE=1 SV=1
J3KSR8	SRSF1	Serine/arginine-rich-splicing factor 1 (Fragment) OS=Homo sapiens OX=9606 GN=SRSF1 PE=1 SV=1
S4R393	ZSWIM8	Zinc finger SWIM domain-containing protein 8 OS=Homo sapiens OX=9606 GN=ZSWIM8 PE=1 SV=1
A0A087WT48	DNAJA2	DnaJ homolog subfamily A member 2 (Fragment) OS=Homo sapiens OX=9606 GN=DNAJA2 PE=1 SV=1
A8MW50	LDHB	L-lactate dehydrogenase (Fragment) OS=Homo sapiens OX=9606 GN=LDHB PE=1 SV=1

E5RHG8	ELOC	Elongin-C (Fragment) OS=Homo sapiens OX=9606 GN=ELOC PE=1 SV=1
Q7Z6U0	TBPL1	TATA box-binding protein-like 1 (Fragment) OS=Homo sapiens OX=9606 GN=TBPL1 PE=1 SV=1
H0YIT0	MTA1	Metastasis-associated protein MTA1 (Fragment) OS=Homo sapiens OX=9606 GN=MTA1 PE=4 SV=1
E9PF88	MTA3	Metastasis-associated protein MTA3 OS=Homo sapiens OX=9606 GN=MTA3 PE=1 SV=1
A0A087X0M4	SLC4A1AP	Kanadaptin OS=Homo sapiens OX=9606 GN=SLC4A1AP PE=1 SV=1
M0QYF9	HOMER3	Homer protein homolog 3 OS=Homo sapiens OX=9606 GN=HOMER3 PE=1 SV=1
Q7Z7A4	PXK	PX domain-containing protein kinase-like protein OS=Homo sapiens OX=9606 GN=PXK PE=1 SV=1

MARCE1
