# Supplementary material for: Polypyrimidine tract binding proteins PTBP1 and PTBP2 associate with distinct proteins and have distinct post-translational modifications in neuronal nuclear extract
Source: PLoS One. 2025 Jun 4;20(6):e0325143. doi: 10.1371/journal.pone.0325143 (PMC12136456; doi:10.1371/journal.pone.0325143)
Supplement: S6 Table — Unique proteins that interact and co-elute with PTBP1 are listed in this table. Proteins identified as unspecifically bound to the Ni2 + beads and carried over during recombinant expression and purification have been removed from this list. (PDF) [file pone.0325143.s009.pdf]

Sppl. Table. 6. Proteins unique to PTBP1 incubated in neuronal WERI nuclear extract.

Accession (PTBP1)	Gene Nam	Description
A0A0A0MSI0	PRDX1	Peroxiredoxin-1 (Fragment) OS=Homo sapiens OX=9606 GN=PRDX1 PE=1 SV=1
P31947	SFN	14-3-3 protein sigma OS=Homo sapiens OX=9606 GN=SFN PE=1 SV=1 adapter protein
P05089	ARG1	Arginase-1 OS=Homo sapiens OX=9606 GN=ARG1 PE=1 SV=2
A0A7I2V4J2	NONO	Non-POU domain-containing octamer-binding protein OS=Homo sapiens OX=9606 GN=NONO PE=1 SV=1
A0A0J9YXX5	PUF60	60 kDa poly(U)-binding-splicing factor (Fragment) OS=Homo sapiens OX=9606 GN=PUF60 PE=1 SV=1
Q14134	TRIM29	Tripartite motif-containing protein 29 OS=Homo sapiens OX=9606 GN=TRIM29 PE=1 SV=2
A0A2C9F2M7	PHGDH	D-3-phosphoglycerate dehydrogenase OS=Homo sapiens OX=9606 GN=PHGDH PE=1 SV=1
D6RAF8	HNRNPD	Heterogeneous nuclear ribonucleoprotein D0 (Fragment) OS=Homo sapiens OX=9606 GN=HNRNPD
P10599	TXN	Thioredoxin OS=Homo sapiens OX=9606 GN=TXN PE=1 SV=3
C9J0D1	H2AZ2	Histone H2A OS=Homo sapiens OX=9606 GN=H2AZ2 PE=1 SV=1
A0A494C189	H2AZ1	Histone H2A OS=Homo sapiens OX=9606 GN=H2AZ1 PE=1 SV=1
Q96KK5	H2AC12	Histone H2A type 1-H OS=Homo sapiens OX=9606 GN=H2AC12 PE=1 SV=3
Q99878	H2AC14	Histone H2A type 1-J OS=Homo sapiens OX=9606 GN=H2AC14 PE=1 SV=3
Q9BTM1	H2AJ	Histone H2A.J OS=Homo sapiens OX=9606 GN=H2AJ PE=1 SV=1
Q16777	H2AC20	Histone H2A type 2-C OS=Homo sapiens OX=9606 GN=H2AC20 PE=1 SV=4
P20671	H2AC7	Histone H2A type 1-D OS=Homo sapiens OX=9606 GN=H2AC7 PE=1 SV=2
P0C0S8	H2AC11	Histone H2A type 1 OS=Homo sapiens OX=9606 GN=H2AC11 PE=1 SV=2
Q93077	H2AC6	Histone H2A type 1-C OS=Homo sapiens OX=9606 GN=H2AC6 PE=1 SV=3
Q7L7L0	H2AW	Histone H2A type 3 OS=Homo sapiens OX=9606 GN=H2AW PE=1 SV=3
Q6FI13	H2AC18	Histone H2A type 2-A OS=Homo sapiens OX=9606 GN=H2AC18 PE=1 SV=3
P04908	H2AC4	Histone H2A type 1-B/E OS=Homo sapiens OX=9606 GN=H2AC4 PE=1 SV=2
Q96QV6	H2AC1	Histone H2A type 1-A OS=Homo sapiens OX=9606 GN=H2AC1 PE=1 SV=3
A0A3B3IS11	H2AC21	Histone H2A OS=Homo sapiens OX=9606 PE=3 SV=1
P16104	H2AX	Histone H2AX OS=Homo sapiens OX=9606 GN=H2AX PE=1 SV=2
A0A0U1RR32	hCG_2039	Histone H2A OS=Homo sapiens OX=9606 GN=hCG_2039566 PE=3 SV=1
Q09666	AHNAK	Neuroblast differentiation-associated protein AHNAK OS=Homo sapiens OX=9606 GN=AHNAK
P25311	AZGP1	Zinc-alpha-2-glycoprotein OS=Homo sapiens OX=9606 GN=AZGP1 PE=1 SV=2
K7ENG2	U2AF2	U2 snRNP auxiliary factor large subunit OS=Homo sapiens OX=9606 GN=U2AF2 PE=1 SV=1
M0R0K9	TRIM28	Transcription intermediary factor 1-beta (Fragment) OS=Homo sapiens OX=9606 GN=TRIM28
O00232	PSMD12	26S proteasome non-ATPase regulatory subunit 12 OS=Homo sapiens OX=9606 GN=PSMD12
P29508	SERPINB3	Serpin B3 OS=Homo sapiens OX=9606 GN=SERPINB3 PE=1 SV=2

P13489	RNH1	Ribonuclease inhibitor OS=Homo sapiens OX=9606 GN=RNH1 PE=1 SV=2
F8WBR5	CALM2	Calmodulin-2 OS=Homo sapiens OX=9606 GN=CALM2 PE=1 SV=1
G3V479	CALM1	Calmodulin-1 OS=Homo sapiens OX=9606 GN=CALM1 PE=1 SV=1
E9PQ96	RPS3	40S ribosomal protein S3 OS=Homo sapiens OX=9606 GN=RPS3 PE=1 SV=1
P60842	EIF4A1	Eukaryotic initiation factor 4A-I OS=Homo sapiens OX=9606 GN=EIF4A1 PE=1 SV=1
E7EQG2	EIF4A2	RNA helicase OS=Homo sapiens OX=9606 GN=EIF4A2 PE=1 SV=1
B7WPG3	HNRNPLL	Heterogeneous nuclear ribonucleoprotein L-like OS=Homo sapiens OX=9606 GN=HNRNPLL
P0DMV9	HSPA1B	Heat shock 70 kDa protein 1B OS=Homo sapiens OX=9606 GN=HSPA1B PE=1 SV=1
P0DMV8	HSPA1A	Heat shock 70 kDa protein 1A OS=Homo sapiens OX=9606 GN=HSPA1A PE=1 SV=1
U3KQI2	MEIS2	Homeobox protein Meis2 (Fragment) OS=Homo sapiens OX=9606 GN=MEIS2 PE=1 SV=1
Q5T6W2	HNRNPK	Heterogeneous nuclear ribonucleoprotein K (Fragment) OS=Homo sapiens OX=9606 GN=HNRNPK
A0A0G2JJZ9	DDX39B	Spliceosome RNA helicase DDX39B (Fragment) OS=Homo sapiens OX=9606 GN=DDX39B PE=1 SV=1
E9PEB5	FUBP1	Far upstream element-binding protein 1 OS=Homo sapiens OX=9606 GN=FUBP1 PE=1 SV=1
P50395	GDI2	Rab GDP dissociation inhibitor beta OS=Homo sapiens OX=9606 GN=GDI2 PE=1 SV=2
M0R3H0	RPS16	40S ribosomal protein S16 OS=Homo sapiens OX=9606 GN=RPS16 PE=1 SV=1
P30041	PRDX6	Peroxiredoxin-6 OS=Homo sapiens OX=9606 GN=PRDX6 PE=1 SV=3
Q6ZSL5	DOCK9	Dedicator of cytokinesis protein 9 OS=Homo sapiens OX=9606 GN=DOCK9 PE=1 SV=1
H3BMH2	RAB11A	Ras-related protein Rab-11A (Fragment) OS=Homo sapiens OX=9606 GN=RAB11A PE=4 SV=1
E9PN91	EEF1D	Elongation factor 1-delta OS=Homo sapiens OX=9606 GN=EEF1D PE=1 SV=1
Q9H0L4	CSTF2T	Cleavage stimulation factor subunit 2 tau variant OS=Homo sapiens OX=9606 GN=CSTF2T PE=1 SV=1
G3XAN0	RPS20	40S ribosomal protein S20 OS=Homo sapiens OX=9606 GN=RPS20 PE=1 SV=1
A0A6Q8PGE6	RAB7A	Ras-related protein Rab-7a OS=Homo sapiens OX=9606 GN=RAB7A PE=1 SV=1
P24534	EEF1B2	Elongation factor 1-beta OS=Homo sapiens OX=9606 GN=EEF1B2 PE=1 SV=3
A0A7P0T9U6	P4HB	Protein disulfide-isomerase OS=Homo sapiens OX=9606 GN=P4HB PE=1 SV=1
P30050	RPL12	60S ribosomal protein L12 OS=Homo sapiens OX=9606 GN=RPL12 PE=1 SV=1
E9PMM9	RPS2	40S ribosomal protein S2 (Fragment) OS=Homo sapiens OX=9606 GN=RPS2 PE=1 SV=1
F2Z393	TALDO1	Transaldolase OS=Homo sapiens OX=9606 GN=TALDO1 PE=1 SV=1
Q96FQ6	S100A16	Protein S100-A16 OS=Homo sapiens OX=9606 GN=S100A16 PE=1 SV=1
G3V3U4	PSMA6	Proteasome subunit alpha type OS=Homo sapiens OX=9606 GN=PSMA6 PE=1 SV=1
Q9H1E1	RNASE7	Ribonuclease 7 OS=Homo sapiens OX=9606 GN=RNASE7 PE=1 SV=2
F8W914	RTN4	Reticulon OS=Homo sapiens OX=9606 GN=RTN4 PE=1 SV=1
M0R268	SNRPA	U1 small nuclear ribonucleoprotein A (Fragment) OS=Homo sapiens OX=9606 GN=SNRPA PE=1 SV=1
P63244	RACK1	Receptor of activated protein C kinase 1 OS=Homo sapiens OX=9606 GN=RACK1 PE=1 SV=3

O15442	MPPD1	Metallophosphoesterase domain-containing protein 1 OS=Homo sapiens OX=9606 GN=MPPED1 PE=1 SV=3
G3V3A4	SNW1	SNW domain-containing protein 1 OS=Homo sapiens OX=9606 GN=SNW1 PE=1 SV=1
P18124	RPL7	60S ribosomal protein L7 OS=Homo sapiens OX=9606 GN=RPL7 PE=1 SV=1
P62280	RPS11	40S ribosomal protein S11 OS=Homo sapiens OX=9606 GN=RPS11 PE=1 SV=3
E9PJ04	SF3B2	Splicing factor 3B subunit 2 (Fragment) OS=Homo sapiens OX=9606 GN=SF3B2 PE=1 SV=8
J3QTA0	WAC	WW domain-containing adapter protein with coiled-coil OS=Homo sapiens OX=9606 GN=WAC PE=1 SV=2
Q5VVQ6	YOD1	Ubiquitin thioesterase OTU1 OS=Homo sapiens OX=9606 GN=YOD1 PE=1 SV=1
H0YDD8	RPLP2	60S acidic ribosomal protein P2 (Fragment) OS=Homo sapiens OX=9606 GN=RPLP2 PE=1 SV=1
A0A5F9ZHN2	ASXL2	Putative Polycomb group protein ASXL2 (Fragment) OS=Homo sapiens OX=9606 GN=ASXL2
P05386	RPLP1	60S acidic ribosomal protein P1 OS=Homo sapiens OX=9606 GN=RPLP1 PE=1 SV=1
P00491	PNP	Purine nucleoside phosphorylase OS=Homo sapiens OX=9606 GN=PNP PE=1 SV=2
F8VUA6	RPL18	60S ribosomal protein L18 (Fragment) OS=Homo sapiens OX=9606 GN=RPL18 PE=1 SV=1
E9PP36	RPL8	60S ribosomal protein L8 OS=Homo sapiens OX=9606 GN=RPL8 PE=1 SV=1
P32320	CDA	Cytidine deaminase OS=Homo sapiens OX=9606 GN=CDA PE=1 SV=2
Q96KR1	ZFR	Zinc finger RNA-binding protein OS=Homo sapiens OX=9606 GN=ZFR PE=1 SV=2
O75635	SERPINB7	Serpin B7 OS=Homo sapiens OX=9606 GN=SERPINB7 PE=1 SV=1
H7C004	SERPINB1C	Serpin B10 (Fragment) OS=Homo sapiens OX=9606 GN=SERPINB10 PE=1 SV=1
P05120	SERPINB2	Plasminogen activator inhibitor 2 OS=Homo sapiens OX=9606 GN=SERPINB2 PE=1 SV=2
P00441	SOD1	Superoxide dismutase [Cu-Zn] OS=Homo sapiens OX=9606 GN=SOD1 PE=1 SV=2
Q99497	PARK7	Parkinson disease protein 7 OS=Homo sapiens OX=9606 GN=PARK7 PE=1 SV=2
F8VZQ9	SARNP	SAP domain-containing ribonucleoprotein OS=Homo sapiens OX=9606 GN=SARNP PE=1 SV=1
V9GYP5	EMG1	Ribosomal RNA small subunit methyltransferase NEP1 (Fragment) OS=Homo sapiens OX=9606
K7EMY3	CHD5	Chromodomain-helicase-DNA-binding protein 5 (Fragment) OS=Homo sapiens OX=9606 GN=CHD5
K7EQH4	ATP5F1A	ATP synthase subunit alpha mitochondrial (Fragment) OS=Homo sapiens OX=9606 GN=ATP5F1A
Q9Y586	MAB21L2	Protein mab-21-like 2 OS=Homo sapiens OX=9606 GN=MAB21L2 PE=1 SV=1
Q5VVC8	RPL11	60S ribosomal protein L11 OS=Homo sapiens OX=9606 GN=RPL11 PE=1 SV=2
D6RD66	WDR1	WD repeat-containing protein 1 (Fragment) OS=Homo sapiens OX=9606 GN=WDR1 PE=1 SV=1
K7EPF6	PGD	Phosphogluconate dehydrogenase (NADP(+)-dependent decarboxylating) OS=Homo sapiens OX=9606
P61019	RAB2A	Ras-related protein Rab-2A OS=Homo sapiens OX=9606 GN=RAB2A PE=1 SV=1
A0A804HJZ5	TBL1X	F-box-like/WD repeat-containing protein TBL1X OS=Homo sapiens OX=9606 GN=TBL1X PE=4 SV=1
Q9BQ87	TBL1Y	F-box-like/WD repeat-containing protein TBL1Y OS=Homo sapiens OX=9606 GN=TBL1Y PE=1 SV=1
Q9Y247	FAM50B	Protein FAM50B OS=Homo sapiens OX=9606 GN=FAM50B PE=1 SV=1
C9J9K3	RPSA	40S ribosomal protein SA (Fragment) OS=Homo sapiens OX=9606 GN=RPSA PE=1 SV=8

P35754	GLRX	Glutaredoxin-1 OS=Homo sapiens OX=9606 GN=GLRX PE=1 SV=2
C9JW69	RCC1	Regulator of chromosome condensation (Fragment) OS=Homo sapiens OX=9606 GN=RCC1 PE=1 SV=1
P48634	PRRC2A	Protein PRRC2A OS=Homo sapiens OX=9606 GN=PRRC2A PE=1 SV=3
A0A7I2V2L0	RPL19	Ribosomal protein L19 OS=Homo sapiens OX=9606 GN=RPL19 PE=1 SV=1
P40429	RPL13A	60S ribosomal protein L13a OS=Homo sapiens OX=9606 GN=RPL13A PE=1 SV=2
A0A087WWR3	HSD11B1L	Hydroxysteroid 11-beta-dehydrogenase 1-like protein OS=Homo sapiens OX=9606 GN=HSD11B1L
P11940	PABPC1	Polyadenylate-binding protein 1 OS=Homo sapiens OX=9606 GN=PABPC1 PE=1 SV=2
O94822	LTN1	E3 ubiquitin-protein ligase listerin OS=Homo sapiens OX=9606 GN=LTN1 PE=1 SV=6
P62851	RPS25	40S ribosomal protein S25 OS=Homo sapiens OX=9606 GN=RPS25 PE=1 SV=1
P39023	RPL3	60S ribosomal protein L3 OS=Homo sapiens OX=9606 GN=RPL3 PE=1 SV=2
A0A494C0M1	MSH6	DNA mismatch repair protein OS=Homo sapiens OX=9606 GN=MSH6 PE=1 SV=1
A0A0A0MRR3	TAF8	TBP-associated factor 8 OS=Homo sapiens OX=9606 GN=TAF8 PE=1 SV=1
P41091	EIF2S3	Eukaryotic translation initiation factor 2 subunit 3 OS=Homo sapiens OX=9606 GN=EIF2S3 PE=1 SV=3
A0A5F9ZGX4	ALDH3A2	Aldehyde dehydrogenase family 3 member A2 (Fragment) OS=Homo sapiens OX=9606 GN=ALDH3A2
P06744	GPI	Glucose-6-phosphate isomerase OS=Homo sapiens OX=9606 GN=GPI PE=1 SV=4

MYH11 S100A16
